# Efficiency of health systems in middle-income countries and determinants of efficiency in Latin America and the Caribbean

**DOI:** 10.1371/journal.pone.0309772

**Published:** 2024-09-05

**Authors:** Laura Goyeneche, Sebastian Bauhoff

**Affiliations:** Social Protection and Health Division, Inter-American Development Bank, Washington, DC, United States of America; The University of Adelaide - North Terrace Campus: The University of Adelaide, AUSTRALIA

## Abstract

We estimate the efficiency of health spending in 145 middle and high-income and the potential gains from improving efficiency for a range of health system outputs using Robust Data Envelopment Analysis for 2010–2014 and 2015–2019 and examine associations with health system characteristics. Focusing on Latin American and Caribbean countries, we find large variability in efficiency and overall substantial potential gains in the later period, despite improvements over time. Our results suggest that, for example, improving spending efficiency could increase life expectancy at birth by 3.5 years (4.6%), or slightly more than the 3.4-year improvement in average life expectancy in the region between 2000 and 2015. Similarly, improved efficiency could reduce neonatal mortality by 6.7 per 1,000 live births (62%), increase service coverage by 6 percentage points (8.7%), and reduce the rich-poor gap in birth attendance by 10 percentage points (12.6%). We find that governance quality is positively associated with efficiency. Overall, the findings indicate an urgent need to improve efficiency in the region and substantial scope for realizing the potential gains of such improvements.

## Introduction

Increasing the efficiency of health spending is a renewed priority for many middle-income countries that are committed to universal health coverage (UHC) and are concerned about the limited fiscal space and financial sustainability of their commitments [1]. On average, Latin American and the Caribbean (LAC) countries have improved on the United Nation’s UHC index from 49.8 in 2000 to 70.9 in 2019 as well as on key health outcomes, such as neonatal mortality and life expectancy [2]. In the same period, per capita health expenditures have increased from US$287 to US$648 [3]. Although spending on health in LAC remains low relative to OECD countries (8.4 percent of GDP relative to 13.9 percent in 2020, respectively), it is projected to increase rapidly [4].

**Fig 1:**
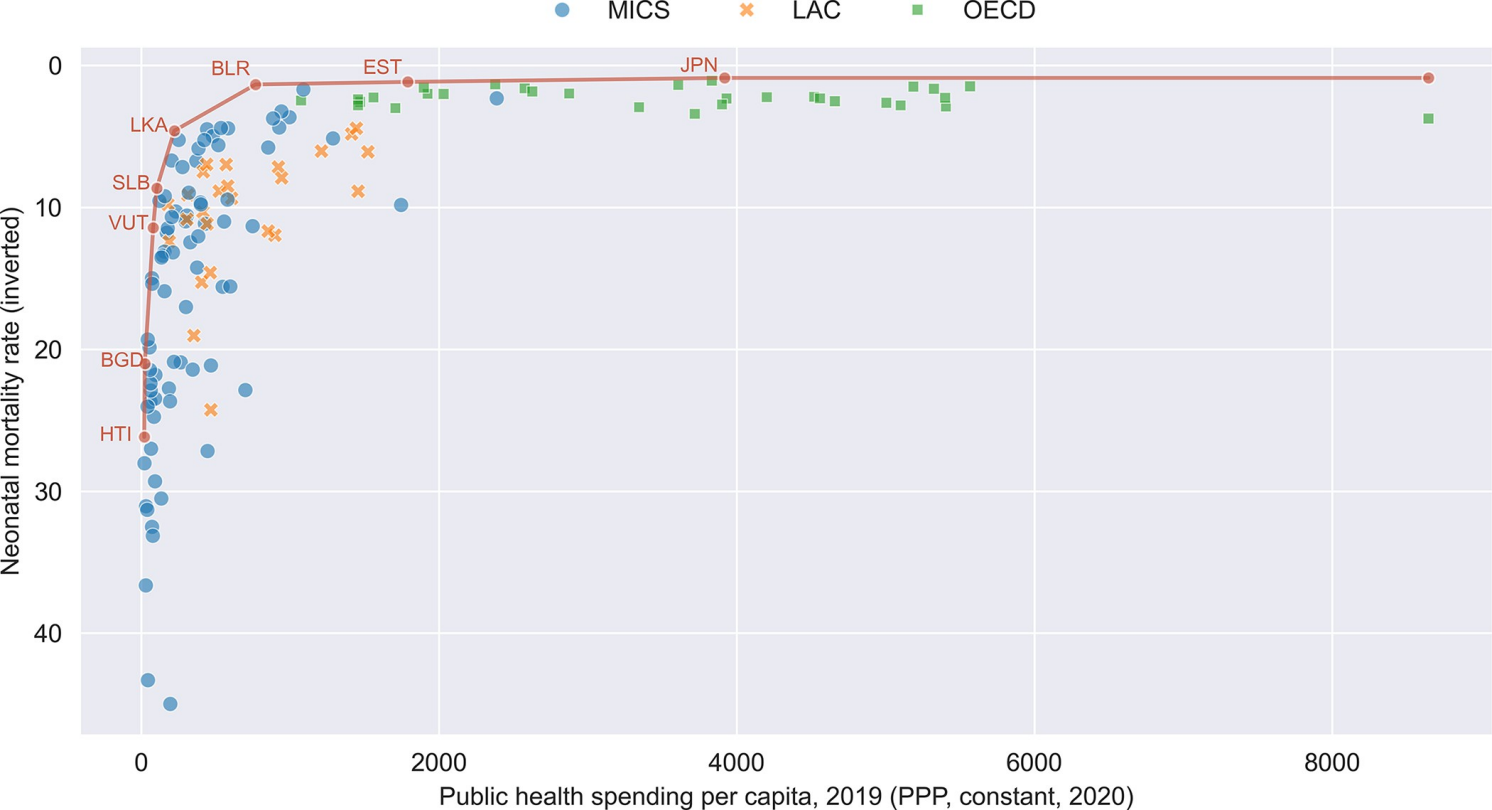
Neonatal mortality and health expenditure per capita efficiency frontier (illustrative). The frontier is drawn for illustration, not derived analytically.

The substantial variation in healthcare outcomes among countries with comparable health expenditures suggests an untapped potential for nations to enhance coverage, outcomes, and equity by improving their spending efficiency. Indeed, existing multi-country studies estimate that 20–40% of health spending may be “wasted” and that low-income countries in particular could advance toward UHC by boosting spending efficiency [5–7]. Fig 1 shows that many countries in LAC could achieve better outcomes with similar per-capita spending if they were as efficient as their peers. For example, Ecuador and Mexico have similar public health spending per capita (approximately US$571 and $581 in 2019) yet their neonatal mortality rates were notably different at 7.0 and 8.51 per 1,000 live births, respectively. Similarly, El Salvador and the Dominican Republic have similar levels of per-capita spending ($439 and $467, respectively) but at 7.0 per 1,000 live births, El Salvador’s neonatal mortality rate is more than three times lower than that of the Dominican Republic (24.3 per 1,000 live births).

In this paper, we use robust data envelopment analysis (DEA) to estimate the efficiency of health spending and the potential gains from improving efficiency in 145 countries–including 26 LAC–in 2010–2014 and 2015–2019 and examine associations of efficiency with health systems characteristics. Our main objective is to identify existing levels of health efficiency, changes over time, and the potential determinants of efficiency across LAC countries. Following prior work [8], we measure a country’s efficiency as the gap between its actual and its possible performance, where the latter reflects how well a country’s “best” peers perform with similar levels of per capita health spending. This measure of technical effiency (the output obtained from a given set of inputs) describes how effectively resources are transformed into health system outputs and health outcomes.

Our analysis makes three main contributions to previous research on countries’ efficiency of health spending. First, we provide updated country-specific estimates of health spending efficiency and potential gains for a larger set of outcomes than in previous studies, including the UHC index. Prior work used DEA to examine several health outcomes and coverage measures for individual LAC countries for the combined period 2011–2015 [8]. Their results show large and variable potential gains, for example, that LAC could gain around five years in life expectancy at birth from improving efficiency but these potential gains range from 1.7 in Costa Rica to 10.4 in Trinidad and Tobago. Building upon this methodology, we include a larger set of output indicators and two periods, 2010–2014 and 2015–2019. Another studyfound similar average potential gains in life expectancy at birth for LAC in 2017 but did not report country-level estimates [9]. Second, we report on country-specific changes in potential gains between the early and late 2010s. This builds on earlier findings that efficiency improved in most countries between 2003–2007 and 2013–2017 without identifying countries [9]. Following this earlier work, we decompose the overall change to examine whether it is due to changes in efficiency (moving closer to the efficiency frontier), technological change (moving with the efficiency frontier as it changes) or a combination of both. Third, we examine both, correlates of efficiency in 2015–2019 and of improvements in efficiency over time. The earlier studies focused on correlates of cross-sectional efficiency and found indicative associations with governance quality in LAC [6,8] and inequality and control of corruption for the group of emerging markets and developing economies [9].

Our results are directly relevant to policy deliberations on increasing the returns to spending on health and expanding the resources available for the health sector [10]. Our analysis suggests that the potential gains from improving efficiency are sizeable: many countries could substantially improve outcomes if they spent their resources with similar efficiency as their peers. The decomposition suggests that most efficiency gains over time were due to technological change rather than more efficient spending. Moreover, our findings indicate that certain institutional features–in particular, governance quality–could help countries improve efficiency and realize these potential gains.

## Methods and data

### Data Envelopment Analysis (DEA)

#### Methodology

We use output-oriented, bias-corrected robust data envelopment analysis (RDEA) to estimate the variable returns-to-scale (VRS) efficiency of health spending in LAC. RDEA is a non-parametric method that for each country, or decision-making unit (DMU), identifies the efficiency frontier consisting of peer countries with the highest performance at each level of spending, accounting for contextual factors. This allows for estimating each country’s efficiency as the distance to the frontier and the potential gains that countries could achieve by moving to the frontier [9,11–13]. RDEA evaluates efficiency relative to actual peer performance, and output-oriented efficiency estimates the required changes in the output value to reach the frontier at the same input level while input-oriented efficiency assesses the changes in the input value required to achieve the same output level in the frontier. Earlier studies find that estimated efficiency scores from output and input-oriented DEAs are highly correlated [9]. By adopting the output-oriented approach, our analysis aims to identify the extent to which countries can improve health outcomes while holding health spending constant. An input-oriented approach would consider how much less countries could spend to maintain the same outputs. Yet in many LMICs, health spending is already low compared to high-income countries, and reducing spending may be less feasible than improving outcomes [6]. We estimate single-output RDEAs because multi-output RDEAs can reduce the ability to discriminate among countries and may assign unreasonable weights to some outputs [8]. Additionally, we estimate single-output models using indexes which capture multiple outputs, such as the UHC index and mortality rate. We conducted the analysis using the DEA implementation in Stata MP 17 [14,15].

The RDEA estimates can under-state the full scope for improvement because RDEA evaluates efficiency relative to the empirical frontier consisting of peer countries’ observed performance rather than a benchmark of full efficiency. In this sense, countries with high RDEA efficiency scores may still be able to improve outcomes with the same spending. Moreover, countries may lie on the efficiency frontier by construction if they do not have peers with comparable spending levels. This can occur in cases where countries have unusually high or low per-capita spending, such as the U.S.A. and Haiti, respectively. As a result, these countries appear highly efficient.

For our analysis and following previous work [8], our analysis sample includes LAC countries as well as OECD and non-LAC middle-income countries (MICs). We consider 18 outputs related to population health status or health outcomes, service coverage, access to services, and equity. These outputs differ in the extent to which they are amenable to health care or the health system. For example, life expectancy at birth depends on many factors outside of the health system, while neonatal mortality and service coverage may be more directly influenced by health systems and policies. Additionally, in line with the Sustainable Development Goals, we consider universal health coverage as a policy goal and therefore an output of the health system. For our main estimations, we run individual DEA models for each of the following outputs:

1. Health outcomes: life expectancy at birth (years), healthy life expectancy at birth (years), the neonatal mortality rate per 1,000 live births, under-5 mortality rate per 1,000 live births, disability-adjusted life years lost (DALYs) for all causes per 100,000 population, DALYs for non-communicable diseases (NCD) per 100,000 population, DALYs for maternal causes per 100,000 population, and DALYs for neonatal causes per 100,000 population.
2. Service coverage: universal health coverage (UHC) service coverage index, including its components: service capacity on access, NCD, reproductive, maternal, newborn, and child health (RMNC), and infectious diseases.
3. Access to services: percentage of skilled birth attendance, and DPT immunization rate (% of children aged 12 to 23 months).
4. Equity: ratio of the poorest/richest wealth quintiles of births attended by skilled health staff, as well as the ratio of rural/urban births attended by skilled health staff.

The main input is public health spending per capita (PPP, constant, 2020) and other inputs (external constraints) are GDP per capita (PPP, constant, 2020) and population aged 65 and above. As Moreno-Serra et al. (2019) note, other possibly relevant inputs–including social and environmental determinants–tend to be highly correlated with our input set.

We present the results potential gains and their percentage increase compared to the baseline and report the estimated efficiency scores in the Supplemental Material. The corresponding potential gains from moving to the frontier are expressed in the units of the output.

### Data

We use data between 2010–2014 and 2015–2019 for a total of 145 countries, consisting of 26 LAC countries, 34 non-LAC OECD countries, and 89 non-LAC middle-income countries (MICs). The data come from the World Bank World Development Indicators (WDI), World Bank Worldwide Governance Indicators, World Health Organization (WHO) Global Health Observatory, WHO Global Health Expenditure Database, and the Institute of Health Metrics and Evaluation (IHME). S1 and S2 Appendices lists the countries and sources for all variables.

We transform the data in two ways. First, to ensure consistency in our analysis, we adopted a “more is better” approach by using the inverse of the percentage of the population aged 65 or more, mortality rates, and burden of disease rates (see S1 Table). Second, we use average values to account for outliers resulting from external shocks, data measurement errors, and missing values in specific years. The data for health outcomes, access to services, and the explanatory variables are averages between 2010–2014 and 2015–2019, respectively; the service coverage is a 2017–2019 and 2010–2015 and the equity measures use the most recent data available.

### Health efficiency over time

To estimate changes in health expenditure efficiency, we compared the RDEA estimates between 2010 to 2014 and 2015 to 2019. First, we compared the estimated efficiency scores between the two time periods to understand the evolution of spending efficiency related to various health outcomes. Second, we compute the Malmquist index to quantify the changes over time driven by efficiency improvement, technological advancements, or a combination of both [9]. We categorize countries into four groups: efficiency and technological improvement (dual gains), efficiency improvement and technological regress (efficiency gain), efficiency loss and technological progress (tech gain), and efficiency and technological losses (dual loss). Countries with efficiency gains showed increased efficiency over time, whereas those with only tech gains improved due to advancements in technology (e.g., improved treatment for prevalent disease conditions) without a corresponding increase in their efficiency scores relative to the most efficient peers. Countries achieving dual gains exhibited both an increase in efficiency and a reduction in the gap with the efficiency frontier (constructed based on high-performing peers), indicating not only advancement in technology, but also significant improvements in their efficiency over time.

### Association with health systems characteristics in LAC

To estimate the associations between efficiency scores for each output indicator and various potential efficiency determinants, we employed the Simar-Wilson cross-sectional regressions. This approach takes into account the bounded nature of RDEA efficiency scores, corrects the standard errors obtained from conventional regression models, and simulates the unknown correlation among efficiency scores while calculating bootstrapped standard errors [8,9,16].

We examine two broad categories of potential determinants of efficiency that are related to the health system. First, as measures of the organization of healthcare financing and delivery, we utilized the out-of-pocket health expenditure as a proportion of the total health expenditure and the number of hospital beds per 1,000 people. Second, as a measure of the quality of governance, we computed an average score based on six governance dimensions derived from the World Bank Worldwide Governance Indicators. These dimensions include government effectiveness, voice and accountability, rule of law, regulatory quality, political stability and absence of violence/terrorism, and control of corruption. We focus on determinants that could be influenced by health policy but note that other studies have found efficiency to be associated with broader characteristics of countries, e.g., income levels, income inequality, and education [9,17].

## Results

### Health system potential gains

**Fig 2:**
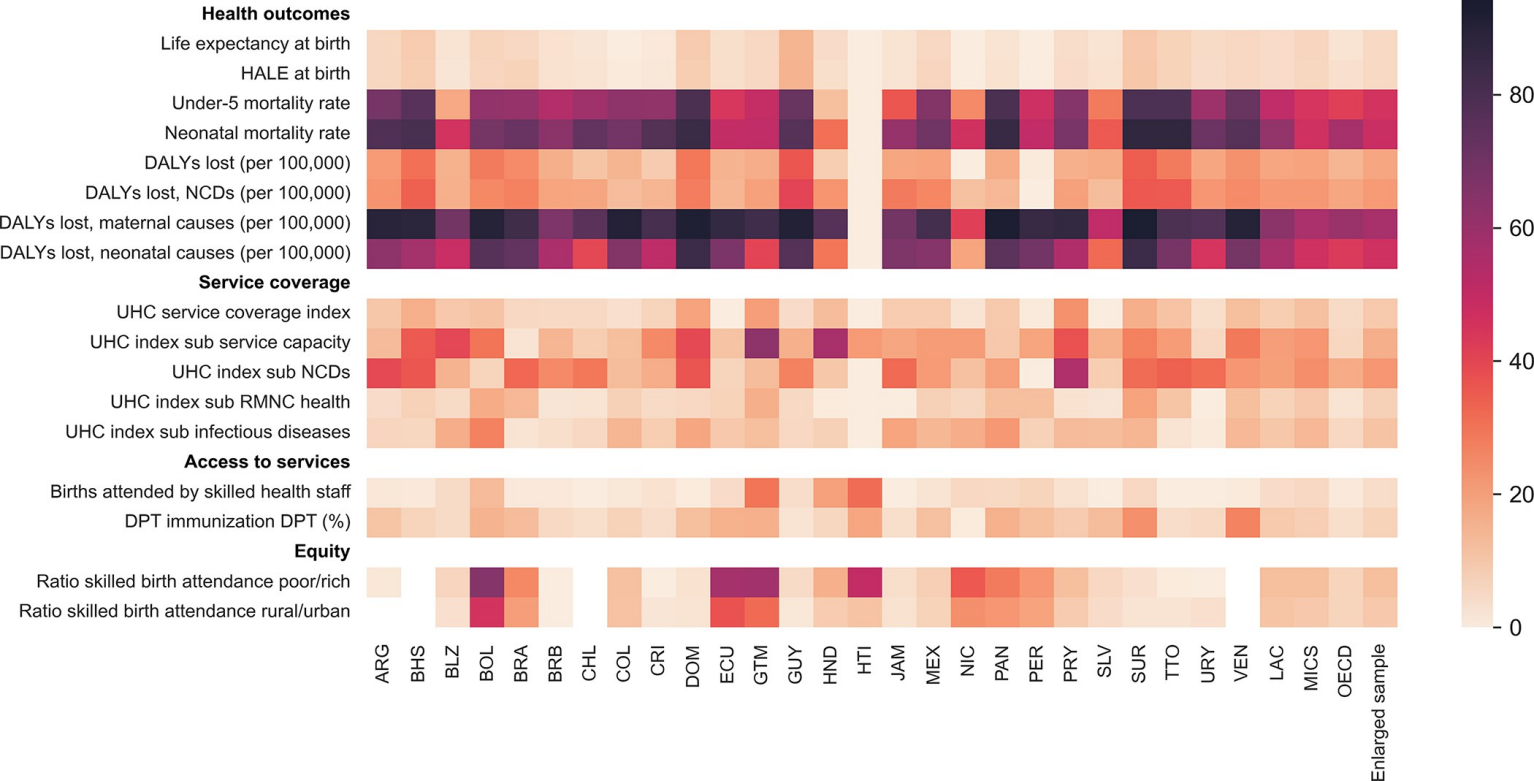
Cross-country variation in potential gains (percent relative to baseline), 2015–2019. Average efficiency scores for MICS and OECD countries include countries in LAC. “Enlarged sample” includes LAC, non-LAC MICS, and non-LAC OECD countries. Results from output-oriented RDEA model using as input variables public health spending per capita, GDP per capita, and population aged 65 and above. See numeric results in S6 Table.

Overall, our analysis revealed significant potential for LAC countries to generate more output for the same per-capita health spending. Table 1 and Fig 2 shows the average potential gains and the variation relative to the baseline that can be achieved across indicators of system performance in different regions (for numeric estimates and confidence intervals see Supplemental Material). These potential gains represent the average improvements in outputs that regions could attain by moving to the estimated efficiency frontier. They reflect the empirical pattern of the efficiency scores.

**Table 1:** Potential gains due to more efficient health spending by country in LAC (2015–2019).

Country	Public health spending per capita	Life expectancy at birth	Neonatal mortality rate	DALYs lost (per 100,000)	UHC service coverage index	Births attended by skilled health staff	Ratio of skilled births attendance poor/rich
ARG	$1,523	4.72	4.82	5,967	7.69	1.67	0.02
BHS	$921	6.61	5.81	9,620	11.16	1.23	--
BLZ	$313	1.92	4.23	4,110	6.64	4.62	0.06
BOL	$406	4.94	10.59	9,048	7.41	11.63	0.23
BRA	$607	4.44	6.78	7,695	4.40	1.30	0.19
BRB	$524	2.50	5.67	5,365	4.04	1.13	0.00
CHL	$1,413	1.53	3.58	2,644	4.22	0.24	--
COL	$943	0.29	5.54	3,673	2.70	1.69	0.11
CRI	$1,210	1.09	4.73	2,129	5.09	2.91	0.00
DOM	$468	6.72	20.68	9,213	12.66	0.38	0.03
ECU	$571	3.06	3.52	3,967	0.00	4.40	0.25
GTM	$188	4.02	6.32	5,022	11.72	21.12	0.24
GUY	$353	10.19	14.77	14,832	3.40	4.07	0.05
HND	$178	3.39	3.04	2,351	7.98	16.03	0.14
HTI*	$20	0.00	0.00	0	0.00	13.40	0.09
JAM	$414	1.85	6.27	5,081	6.33	0.30	0.04
MEX	$581	4.34	6.01	5,294	6.47	2.50	0.08
NIC	$306	0.09	5.10	0	1.92	5.41	0.23
PAN	$1,457	2.20	7.59	4,055	7.43	5.11	0.21
PER	$417	0.00	3.80	233	0.77	6.39	0.17
PRY	$442	3.33	7.63	4,010	14.36	3.19	0.11
SLV	$439	1.73	2.50	5,115	0.00	0.04	0.05
SUR	$898	7.26	10.50	11,820	11.34	5.46	0.04
TTO	$853	5.45	10.24	9,419	8.08	0.00	0.00
URY	$1,445	3.71	3.17	5,689	2.98	0.02	0.00
VEN	$464	4.21	11.31	6,806	8.85	0.89	--
**LAC**	$667	3.45	6.70	5,506	6.06	4.43	0.10
**MICS**	$395	4.58	6.93	6,854	7.36	4.99	0.10
**OECD**	$3,201	2.25	1.59	4,288	3.91	1.20	0.06
**Total**	$1,161	4.00	5.56	6,238	6.50	3.97	0.09

For the complete table of efficiency scores for all output indicators considered, see Supplemental Material. Results from output-oriented RDEA model using as input variables public health spending per capita, GDP per capita, and population aged 65 and above. Public health spending per capita (PPP, constant, 2020) corresponds to the 2015–2019 average value. * Haiti’s spending is uniquely low and therefore on the efficiency frontier by construction, for many outputs.

Relative to baseline values, the LAC region could improve life expectancy at birth by 4.6 percent (3.45 years) and increase the overall UHC index by 8.7 percent (6 percentage points). Similarly, the region could reduce neonatal mortality by 62 percent (6.7 per 1,000 live births), and reduce the rich-poor gap in birth attendance by 12.6 percent (10 percentage points). The relative potential gains for DALYs lost to maternal and neonatal causes are higher than the gains for DALYs lost due to NCDs. The reverse holds for coverage, where the potential gains for NCDs are relatively larger than for reproductive, maternal, newborn, and child health services. As a region and as a percent of baseline output, LAC generally has lower potential gains than non-LAC MICs and higher potential gains than OECD countries, reflecting that the latter has higher efficiency (Fig 2 and S5 Table). For example, the OECD and non-LAC MICs could improve life expectancy at birth by 2.8 percent and 6.5 percent respectively. LAC has similar or more potential gains as other MICs with regards to DAYLs lost and equity-in-access.

There is substantial heterogeneity in the efficiency of health spending within LAC, across countries and outputs. On average, fewer than 30 percent of LAC countries have lower potential gains (relative to baseline) than observed in OECD countries and 47 percent outperformed the average potential gains of non-LAC MICs. El Salvador, Barbados, Chile, and Costa Rica have lower relative potential gains than the LAC average for at least 13 of the 17 indicators assessed. For life expectancy and HALE at birth, Haiti, Peru, and Nicaragua showed the lowest potential gains. El Salvador, Haiti, Ecuador, Peru, Nicaragua, and Colombia had the smallest potential to improve the overall coverage index, relative to their baselines. Meanwhile, Costa Rica and Barbados had the smallest relative potential gains to improve on the equity measures.

Table 1 shows country-level potential gains for selected outputs. Overall, Bolivia, Dominican Republic, Guatemala, Guyana, and Suriname have the largest potential gains for seven or more of the 17 indicators assessed, exhibiting substantial opportunities for improvement. Among health outcomes, in the Dominican Republic, Guyana, and Suriname, life expectancy at birth could increase by between 6.7 to 10.2 years (an increase between 10 to 15 percent). For service coverage, the UHC index shows a potential increase of up to 11.7 to 14.4 percentage points in the Dominican Republic, Guatemala, and Paraguay, with a greater potential for the service capacity and NCDs sub-index of up to 20 percentage points (S5 Table). In the Dominican Republic, Guyana, and Suriname, neonatal mortality could be reduced by between 10.5 to 20.7 deaths per 1,000 live births, a reduction between 78 to 88 percent. For access to services outputs, the largest potential gains are observed in Guatemala and Venezuela, with a potential increase of 21.1 percentage points in the skilled birth attendance rate and a potential increase of 19.5 percentage points in DPT immunization, respectively. The ratios in skilled birth attendance could also be reduced by 20 percentage points for the poor/rich and rural/urban gap in Bolivia, Ecuador, and Guatemala. Haiti has the lowest potential gains (highest efficiency scores) for its per-capita spending, including zero potential gains or most outputs, indicating that it lies on the efficiency frontier.

### Evolution of health efficiency over time

**Fig 3:**
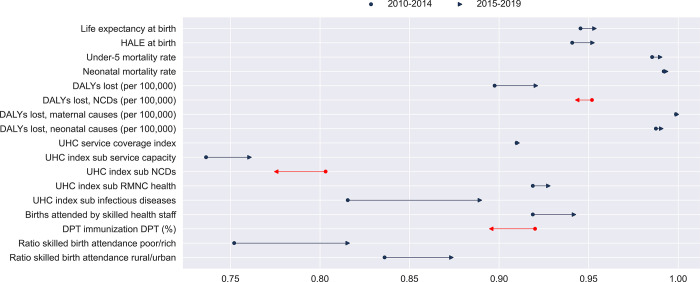
Change in efficiency score in LAC countries, 2010–2014 vs. **2015–2019.** Average efficiency scores for the 26 countries in LAC.

LAC experienced improvements in efficiency for most outputs between 2010 to 2014 and 2015 to 2019. 14 of the 17 outputs had higher efficiency scores in the later period (Fig 3, for numeric estimates, see S2 and S4 Tables). The largest improvements are for the UHC sub-index for infectious diseases, as well as the ratio for skilled birth attendance for the poor/rich and rural/urban, with increases of 7.5, 6.3, and 3.7 percentage points, respectively. No significant changes were observed in DALYs lost for maternal causes and the UHC index. The UHC sub-index for NCDs and the DPT immunization rate, on the other side, showed a decrease of 2.8 and 2.4 percentage points, respectively.

Country-specific results show that Colombia, Ecuador, Honduras, and Haiti demonstrated improvements in efficiency in between 13 to 16 outputs. Colombia showed large improvements in the UHC sub-index for infectious diseases (26 percentage points) and service capacity (5.3 percentage points) but experienced a decline of 2 percentage points in the UHC sub-index for NCDs. Ecuador exhibited the largest increase in the UHC sub-index for infectious diseases (19 percentage points) and skilled birth attendance (4.3 percentage points). Honduras demonstrated progress in the ratios of skilled birth attendance for the poor/rich (33.2 percentage points) and rural/urban (13.8 percentage points), but experienced a decline in the UHC sub-index for NCDs, skilled birth attendance, and DPT immunization, by 2.2, 3.3, and 5.8 percentage points, respectively. As noted above, Haiti defines the frontier because it is unique at its level of spending, so the results need to be interpreted cautiously. With this caveat, Haiti saw an increase in most indicators except for the UHC sub-index for NCDs, which remained unchanged.

In contrast, Uruguay, Bahamas, Suriname, Dominican Republic, and Mexico showed improvements in fewer than 7 outputs, with declines in the UHC index, the UHC sub-index for NCDs, and DPT immunization. Venezuela experienced a decrease in spending efficiency in all 17 outputs. Haiti showed a 12.3 percentage point increase in DPT immunization efficiency, while Jamaica, Chile, Colombia, Trinidad and Tobago, Barbados, and Costa Rica also saw improvements ranging from 2 to 5 percentage points. In contrast, Venezuela experienced a decline of 27 percentage points, followed by Brazil, Guatemala, and Bolivia with declines of 10.5 and 7 percentage points, respectively. In terms of DALYs lost for NCDs, while Haiti saw an increase of 8.7 percentage points in efficiency scores, Venezuela, Suriname, the Dominican Republic, Uruguay, Jamaica, and Mexico presented decreases of 2 to 6 percentage points. The remainder of the countries exhibited smaller declines in NCD outcomes, with changes of less than or equal to one percentage point.

**Fig 4:**
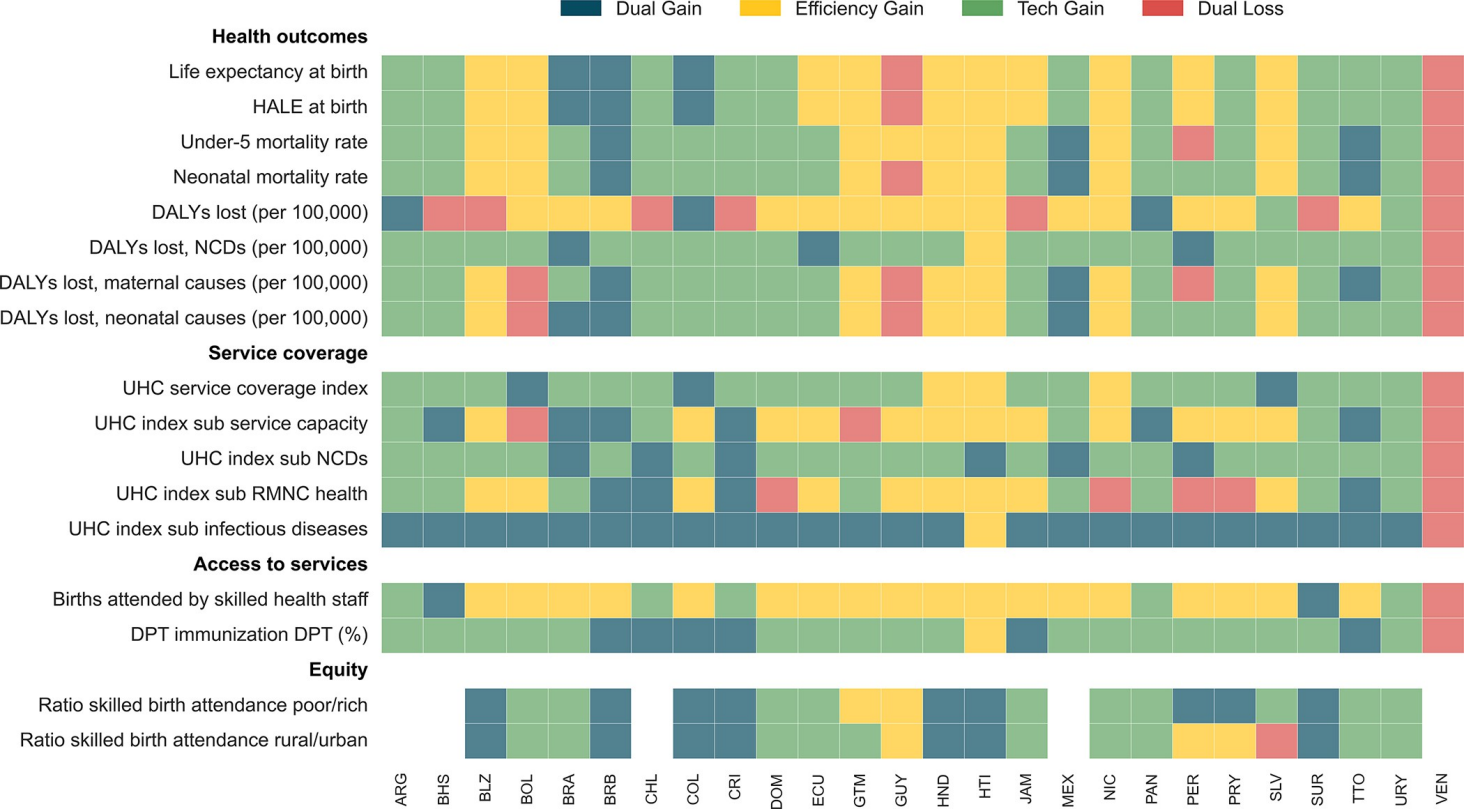
Change in outcomes driven by efficiency and technology in LAC countries, 2010–2014 vs 2015–2019. Output-oriented decomposition of Malmquist Index. Dual gains refer to improvements in both efficiency and technology. Efficiency gain denotes improvement in efficiency despite a technological regression. Tech gain indicates improvements in technology accompanied by efficiency losses. Dual loss corresponds to declines in both efficiency and technology. See results for the enlarged sample in S4 Fig.

Turning to the drivers of the overall changesresults from the decomposition suggest that health systems in the region experienced significant changes in efficiency and technology between 2010 to 2014 and 2015 to 2019 (Fig 4). Life expectancy presented dual gains in efficiency and technology in Brazil, Barbados, and Colombia. The remaining countries, except Venezuela, presented partial improvements in either dimension. Regarding DALYs lost to NCDs, most countries presented technological advances despite efficiency losses. Brazil, Ecuador, and Peru are exceptions, with improvements in both areas. Additionally, under-5 and neonatal mortality rates in Barbados, Mexico, and Trinidad and Tobago saw significant dual gains. The UHC service coverage index revealed only technological progress in 19 countries, with Bolivia, Colombia, and El Salvador achieving improvements in both efficiency and technology. In the UHC sub-index for infectious diseases, all countries except Haiti and Venezuela made efficiency gains and technological progress. In access to services indicators, some countries achieved dual gains, although partial gains attributed to either efficiency or technology were more common. Finally, equity indicators revealed dual gains in Belize, Barbados, Colombia, Costa Rica, Honduras, Haiti, and Suriname, with El Salvador being the only country to exhibit a decline in efficiency and technology in its equity outcomes.

### Potential determinants of health efficiency

Table 2 reports estimates of the correlation between the estimated RDEA efficiency scores for all outputs and a set of potential efficiency determinants. We observed significant associations between the health system organization and governance quality indicators and efficiency scores (for further details see S9 Table). Focusing on the enlarged sample and with the caveat that these are not causal estimates, we found that governance quality is positively associated with most outputs, particularly health and service coverage outcomes. Our estimations indicate that a one-unit increase in the average governance quality is associated with an improvement of 0.058 and 0.088 in the efficiency scores for DALYs lost per 100,000 people and the UHC coverage index, respectively. This translates to a reduction of 7,076DALYs and an increase of 10.7 percentage points in the coverage index if LAC achieved the OECD-average governance quality for the same level of public health spending. When we examined the individual components of the governance quality index, we found that the association is primarily driven by measures of government effectiveness and political stability; however, these analyses have limited statistical power (S10 Table). Furthermore, the number of hospital beds per 1,000 people is negatively associated with efficiency for health outcomes, but positively associated with coverage indices, access (skilled birth attendance), and the ratio in skilled birth attendance for the poor/rich and urban/rural areas.

**Table 2:** Potential determinants of efficiency, 2015–2019 cross-section.

	Life expectancy at birth	Neonatal mortality rate	DALYs lost per 100,000 people	UHC services coverage index	Births attended by skilled health staff	Ratio skilled birth attendance poor/rich
	(1)	(2)	(3)	(4)	(5)	(6)
OOP health expenditure as % of CHE	0.000	0.000	0.001	0.001	-0.003	0.009
	(0.000)	(0.000)	(0.001)	(0.001)	(0.006)	(0.010)
Hospital beds per 1.000 people	-0.002	0.005*	-0.009*	0.021*	0.824***	0.602
	(0.003)	(0.002)	(0.005)	(0.011)	(0.278)	(0.400)
Average governance quality	0.033***	0.010**	0.058***	0.088***	0.777***	0.217
	(0.011)	(0.005)	(0.020)	(0.033)	(0.314)	(0.430)
Constant	0.960***	0.994***	0.922***	0.852***	1.284***	0.331
	(0.023)	(0.007)	(0.034)	(0.055)	(0.422)	(0.439)
Observations	105	107	106	102	86	57
Numer of efficiencient DMUs	3	13	14	18	18	12
Model degress of freedom	3	3	3	3	3	3
Model chi-squared	9.32	5.88	9.215	10.821	9.15	2.452
Model significance, p-value	0.003	0.118	0.027	0.013	0.027	0.484

Simar-Wilson models estimated with 1,000 bootstrap replications. Robust standard errors in parenthesis. *p<0.1, **p<0.5, **p<0.01. Results using enlarged sample, which includes LAC, non-LAC MICS, and non-LAC OECD countries. Pearson correlations between variables are presented in S1 Fig, and additional model results are available in S9 Table.

In addition to the results with the enlarged sample, we further explored a model that included only the 26 countries in the LAC region. We found no significant associations between these indicators and efficiency scores for health and equity outcomes (S9 Table). For service coverage, we found that higher shares of OOP expenditures (for infectious diseases) and higher governance quality (for RMNCH) are associated with higher efficiency in the service coverage sub-indices for RMNCH and infectious diseases. Similarly, there is preliminary indication that better governance is associated with higher efficiency in providing access to necessary services such as DTP immunization.

### Sensitivity analysis

To assess the robustness of the findings, we estimated RDEA models using total, public, and pooled health spending per capita only, and total and pooled health spending instead of public health spending per capita with the external constraints (GDP per capita and population aged 65 and above) as inputs. We obtained similar results from a model with only health spending, suggesting that adding more inputs may not qualitatively affect the main findings (see S2 and S3 Figs; S7 and S8 Tables). These sensitivity tests did not produce substantively different results from our main model.

We extended the sensitivity analysis by estimating a multi-output RDEA model incorporating life expectancy and the UHC index as outputs, and public health spending per capita with external constraints as inputs. We found a slight improvement in the average efficiency scores when estimating the two-output model compared to the single-output model in the same outputs, with efficiency scores increasing by 1.1 points for life expectancy and 6.6 points for the UHC index. In addition, we found a high rank correlation between the single and two-output models, with correlations of 0.9 for life expectancy and 0.8 for the UHC index. This suggests that incorporating multiple outputs may not substantively change the conclusions drawn from our single-output model analyses.

## Discussion

We find that across LAC countries, improving health spending efficiency could produce large gains in health outcomes, service coverage, and equity. For example, by moving vertically to the empirical efficiency frontier, the average country in LAC could gain about 3.5 more life years at birth (4.6 percent), increase overall UHC service coverage by 6 percentage points (8.7 percent), and reduce poor/rich gap in skilled birth attendance by 10 percentage points (12.6 percent). Efficiency is low with regard to service coverage for NCDs and equity in the delivery of primary health care services, such as skilled birth attendance, and with regards to preventable disease burden. As a group, LAC countries relative to other regions, have middling spending efficiency: lower than the OECD and higher than non-LAC MICs. Spending efficiency appears to have improved slightly since the 2010s in LAC, and higher efficiency is correlated with governance quality.

Together with earlier research, our results highlight the urgent need for LAC and MICs to improve spending efficiency. For context, average life expectancy at birth in LAC improved by 3.4 years between 2000 and 2015, that is, the potential gains of 3.5 years from improving spending efficiency in the region are comparable to 15 years of progress. While all regions could improve efficiency, the scope in LAC and MICs is larger than in higher-income settings. For example, our analysis suggests that, as a group, OECD countries could gain 2.8 percent of life expectancy at birth compared to 4.6 percent in LAC and 6.5 percent in MICs.

Moreover, while efficiency appears to have improved over time, highlighted by health system efficiency and technology gains on infectious diseases, there is substantial scope for further improvement. This is particularly true for outputs that are core challenges for LAC, including NCDs, where most countries benefitted from technological advances, and access to services, where the achievements vary significantly among countries. These topics, along with the mixed results for UHC service coverage, deserve broader attention. For instance, while Bolivia, Colombia, and El Salvador showed improvements in both efficiency and technology in UHC, the overall progress indicates that achieving universal health coverage [18] requires a more integrated approach that blends technological advancements and health spending efficiency gains.

Our analysis does not support reducing or maintaining spending on health in LAC countries. OECD countries spend more and have better outcomes, which suggests that increasing spending in LAC could lead to improvements in outcomes. The policy challenge, therefore, lies in ensuring that current and future spending are as efficient as possible.

Countries can deploy a range of specific policies to improve technical efficiency [19–21]. This includes disinvesting in technologies and health care services with no or low value; reducing clinical, operational, and admin waste [19]; promoting generic drugs; improving procurement processes and using strategic purchasing; shifting toward output-based payment systems; strengthening managerial capacity; reducing fragmentation; using digital tools where appropriate; tackling corruption; and improving accountability. In addition, countries should improve public financial management in the health sector, which can help translate efficiency gains into fiscal space for health [10].

Our analysis shares the limitations noted in earlier work [8,9]. First, the RDEA efficiency frontier is constructed based on observed performance. We may therefore over-estimate countries’ efficiency. Related, for some countries there are no comparable peers, so the country attains the efficiency frontier by construction. This is the case of Haiti, which has uniquely low per-capita spending in LAC and appears to be highly efficient, which needs to be interpreted cautiously. Excluding Haiti does not affect our main conclusions for the other countries (S3 and S5 Tables). Second, the RDEA is sensitive to transformations of inputs and outputs, particularly when dealing with undesirable outputs transformed using a non-linear function [22]. This is the case for outputs such as mortality, for which we used the inverse in the calculations. In such cases, the efficiency scores show minimal variations across countries and regions. This issue does not affect the estimated potential gains, which is our focus. Third, the simulated potential gains from policy actions are based on associations of potential determinants with outputs that may not represent causal relationships. This analysis is also limited by the availability of global data on determinants of health system characteristics. Fourth, there may be additional contextual factors that affect how effectively spending can be translated into outcomes and that are not captured in our analysis. Fifth, there are additional health systems outcomes—such as financial risk protection and responsiveness–that we did not consider and would appear as inefficiencies in our analysis if they did not contribute to our set of outcomes. Finally, there may be differences in how data on spending and outputs are captured across countries.

Our analysis focused on the macro efficiency of health spending and should be complemented with evidence on the levels and determinants of efficiency at the micro and meso levels, e.g., for hospitals and clinics or the provision of NCD care. Moreover, future research could investigate in more detail the patterns of efficiency over time, e.g., the overall worsening of efficiency for NCDs and DTP (Fig 3) and why some countries maintained or improved efficiency for these outputs.

## Supporting information

S1 Fig Correlation chart. (PDF)

S2 Fig Comparison of efficiency scores due to efficient health spending by model in LAC, 2015–2019. (PDF)

S3 Fig Comparison of potential gains due to efficient health spending by model in LAC, 2015–2019. (PDF)

S4 Fig Change in outcomes driven by efficiency and technology, 2010–2014 vs 2015–2019. (PDF)

S1 Table Input and output indicators. (PDF)

S2 Table Sample averages by country, 2015–2019. (PDF)

S3 Table Efficiency score by output indicator, 2015–2019. (PDF)

S4 Table Efficiency score by output indicator, 2010–2014. (PDF)

S5 Table Potential gains due to efficient health spending by output indicator, 2015–2019. (PDF)

S6 Table Potential gains due to efficient health spending by output indicator (percent relative to baseline), 2015–2019. (PDF)

S7 Table Comparison of efficiency scores due to efficient health spending by model and country for selected output indicators, 2015–2019. (PDF)

S8 Table Comparison of potential gains due to efficient health spending by model and country for selected output indicators, 2015–2019. (PDF)

S9 Table Potential determinants of efficiency, 2015–2019. (PDF)

S10 Table Potential determinants of efficiency with disaggregated analysis by governance quality, 2015–2019. (PDF)

S1 Appendix Sample of countries. (PDF)

S2 Appendix Detailed model results. (ZIP)

## References

[1] Glassman A, Madan Keller J, Smitham E. The Future of Global Health Spending Amidst Multiple Crises [Internet]. Center for Global Development; 2023 [cited 2024 Feb 26]. Available from: https://www.cgdev.org/publication/future-global-health-spending-amidst-multiple-crises.

[2] World Health Organization (WHO). UHC service coverage index [Indicator] [Internet]. [cited 2024 Feb 13]. Available from: https://data.who.int/indicators/i/9A706FD.

[3] World Health Organization (WHO). Global Health Expenditure Database [Internet]. [cited 2024 Feb 13]. Available from: https://apps.who.int/nha/database.

[4] Rao KD, Vecino Ortiz AI, Roberton T, Lopez Hernandez A, Noonan C. Future Health Spending in Latin America and the Caribbean: Health Expenditure Projections & Scenario Analysis [Internet]. Inter-American Development Bank; 2022 [cited 2022 Jun 7]. Available from: https://publications.iadb.org/en/node/32029.

[5] Chisholm D, Evans DB. Improving Health System Efficiency as a Means of Moving Towards Universal Coverage [Internet]. WHO; 2010 [cited 2024 Feb 26]. Report No.: World Health Report (2010) Background Paper 28. Available from: https://cdn.who.int/media/docs/default-source/health-financing/technical-briefs-background-papers/whr-2010-background-paper-28.pdf.

[6] Jordi E, Pley C, Jowett M, Abou Jaoude GJ, Haghparast-Bidgoli H. Assessing the efficiency of countries in making progress towards universal health coverage: a data envelopment analysis of 172 countries. BMJ Glob Health. 2020 Oct;5(10):e002992. doi: 10.1136/bmjgh-2020-002992 33115858 PMC7594203

[7] WHO. World Health Report 2010 [Internet]. World Health Organization; 2010 [cited 2024 Mar 4]. Available from: https://www.who.int/publications-detail-redirect/9789241564021.

[8] Moreno-Serra R, Anaya-Montes M, Smith PC. Potential determinants of health system efficiency: Evidence from Latin America and the Caribbean. PLOS ONE. 2019;14(5):e0216620. doi: 10.1371/journal.pone.0216620 31075148 PMC6510473

[9] Garcia-Escribano M, Mogues T, Juarros P. Patterns and Drivers of Health Spending Efficiency. IMF Work Pap. 2022 Mar;2022(048):1.

[10] Barroy H, Cylus J, Patcharanarumol W, Novignon J, Evetovits T, Gupta S. Do efficiency gains really translate into more budget for health? An assessment framework and country applications. Health Policy Plan. 2021 Oct 1;36(8):1307–15. doi: 10.1093/heapol/czab040 33855342 PMC8428602

[11] Jacobs R, Smith PC, Street A. Measuring Efficiency in Health Care: Analytic Techniques and Health Policy. Cambridge University Press; 2006. 275 p.

[12] Cylus J, Papanicolas I, Smith PC, editors. Health system efficiency: How to make measurement matter for policy and management [Internet]. Copenhagen (Denmark): European Observatory on Health Systems and Policies; 2016 [cited 2022 Nov 18]. (European Observatory Health Policy Series). Available from: http://www.ncbi.nlm.nih.gov/books/NBK436888/. 28783269

[13] Cook WD, Seiford LM. Data envelopment analysis (DEA)–Thirty years on. Eur J Oper Res. 2009 Jan 1;192(1):1–17.

[14] Ji Y bae, Lee C. Data Envelopment Analysis. Stata J Promot Commun Stat Stata. 2010 Jul;10(2):267–80.

[15] StataCorp. Stata Statistical Software: Release MP 17. College Station, TX; 2022.

[16] Simar L, Wilson P. Estimation and inference in two-stage, semi-parametric models of production processes. J Econom. 2007;136(1):31–64.

[17] Greene W. Distinguishing between heterogeneity and inefficiency: stochastic frontier analysis of the World Health Organization’s panel data on national health care systems. Health Econ. 2004;13(10):959–80. doi: 10.1002/hec.938 15455464

[18] Lozano R, Fullman N, Mumford JE, Knight M, Barthelemy CM, Abbafati C, et al. Measuring universal health coverage based on an index of effective coverage of health services in 204 countries and territories, 1990–2019: a systematic analysis for the Global Burden of Disease Study 2019. The Lancet. 2020 Oct 17;396(10258):1250–84. 10.1016/S0140-6736(20)30750-9 PMC7562819 32861314

[19] OECD. Tackling Wasteful Spending on Health [Internet]. 2017 [cited 2023 Aug 30]. Available from: https://read.oecd-ilibrary.org/social-issues-migration-health/tackling-wasteful-spending-on-health_9789264266414-en.

[20] Savedoff W, Gongora P, Giedion U, Distrutti M. Smart Spending for Health: How to Make Each Dollar Count. Inter-American Development Bank; 2023.

[21] Yip W, Hafez R. Improving Health System Efficiency. WHO; 2015.

[22] Zhou Z, Xu G, Wang C, Wu J. Modeling undesirable output with a DEA approach based on an exponential transformation: An application to measure the energy efficiency of Chinese industry. J Clean Prod. 2019 Nov 1;236:117717.

