# Supplementary figures and images for: Efficiency of health systems in middle-income countries and determinants of efficiency in Latin America and the Caribbean
Source: PLoS One. 2024 Sep 5;19(9):e0309772. doi: 10.1371/journal.pone.0309772 (PMC11376550; doi:10.1371/journal.pone.0309772)

**S1 Fig.** Correlation chart

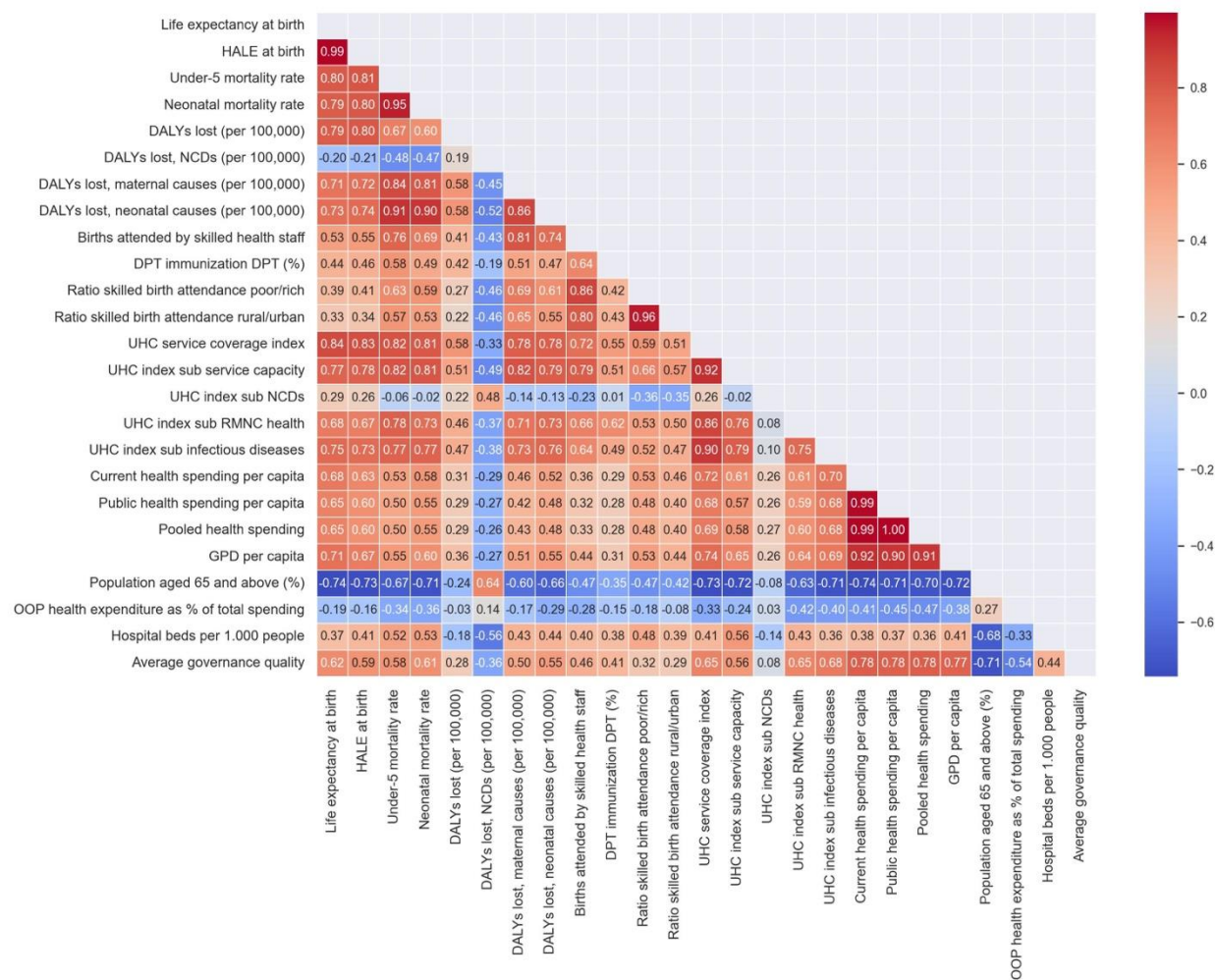

Source: Author's calculations.

Supplement: S1 Fig — (PDF) [file pone.0309772.s001.pdf]
