# Supplementary material for: Efficiency of health systems in middle-income countries and determinants of efficiency in Latin America and the Caribbean
Source: PLoS One. 2024 Sep 5;19(9):e0309772. doi: 10.1371/journal.pone.0309772 (PMC11376550; doi:10.1371/journal.pone.0309772)
Supplement: S2 Fig — (PDF) [file pone.0309772.s002.pdf]

S2 Fig. Comparison of efficiency scores due to efficient health spending by model in LAC, 2015-2019

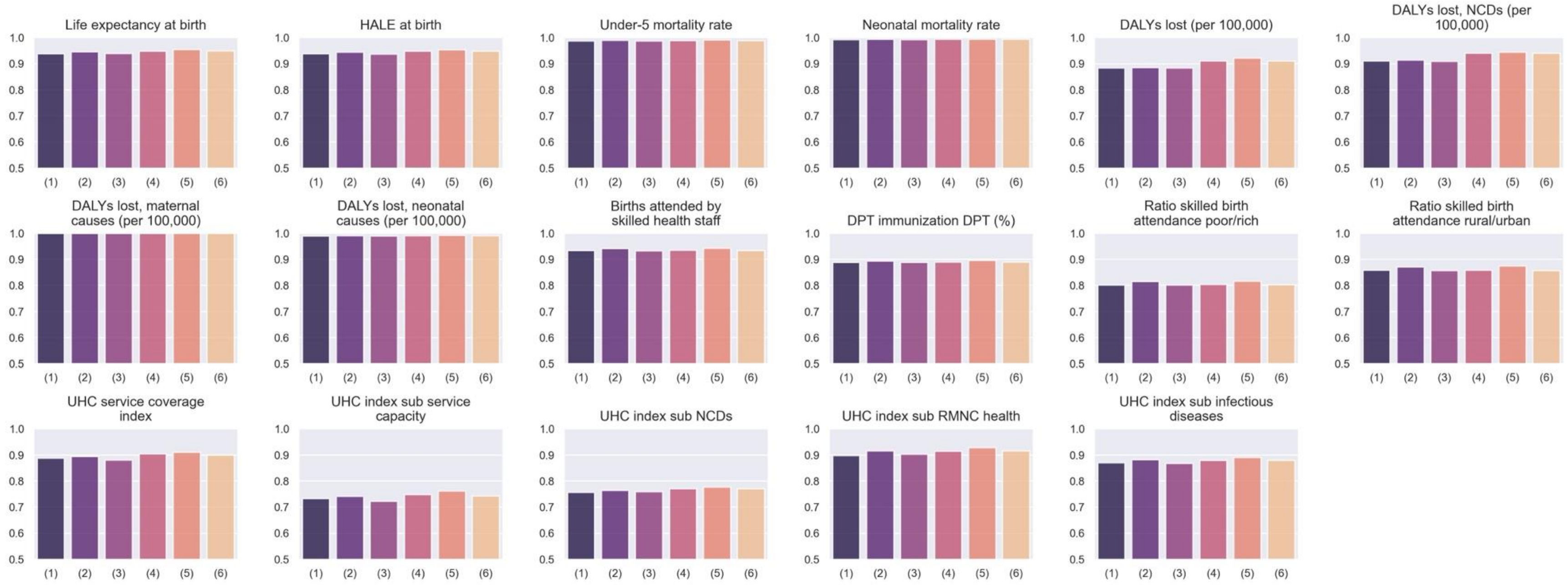

Source: Author’s calculations.

Notes: Fig C1 presents the efficiency score for output-oriented DEA models using different input variables. Model (1) use as input the total health expenditure per capita. Model (2) use as input the total public health expenditure per capita. Model (3) use as input the total public and private health expenditure per capita. Model (4) use as input the total health expenditure per capita, GPD per capita, and population aged 65 and above. Model (5) use as input the total public health expenditure per capita, GPD per capita, and population aged 65 and above. Model (6) use as input the total public and private health expenditure per capita, GPD per capita, and population aged 65 and above.
