# Supplementary material for: Efficiency of health systems in middle-income countries and determinants of efficiency in Latin America and the Caribbean
Source: PLoS One. 2024 Sep 5;19(9):e0309772. doi: 10.1371/journal.pone.0309772 (PMC11376550; doi:10.1371/journal.pone.0309772)
Supplement: S4 Fig — (PDF) [file pone.0309772.s004.pdf]

S4 Fig. Change in outcomes driven by efficiency and technology, 2010-2014 vs 2015-2019

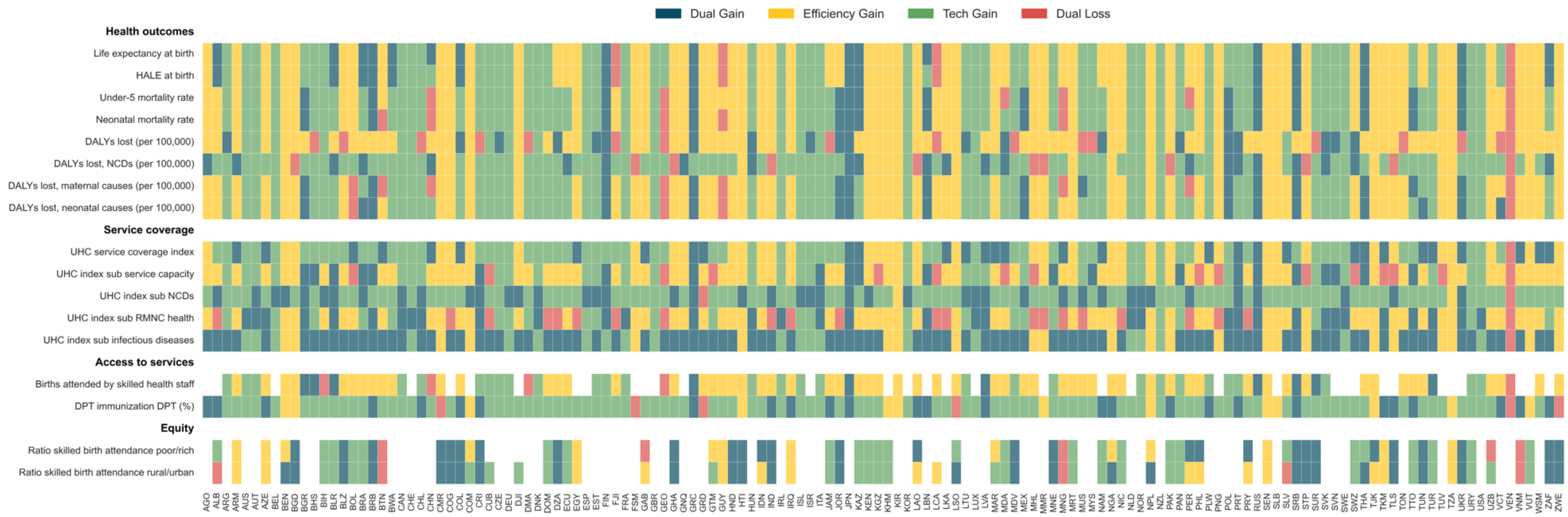

Source: Author's calculations.

Notes: Output-oriented decomposition of Malmquist Index. Dual gains refer to improvements in both efficiency and technology. Efficiency gain denotes improvement in efficiency despite a technological regression. Tech gain indicates improvements in technology accompanied by efficiency losses. Dual loss corresponds to declines in both efficiency and technology.
