# Supplementary material for: Efficiency of health systems in middle-income countries and determinants of efficiency in Latin America and the Caribbean
Source: PLoS One. 2024 Sep 5;19(9):e0309772. doi: 10.1371/journal.pone.0309772 (PMC11376550; doi:10.1371/journal.pone.0309772)
Supplement: S1 Table — (PDF) [file pone.0309772.s005.pdf]

S1 Table. Input and output indicators

Variables	Year	Source
**Input variables**		
Population aged 65 and more (% of total) *	2015-19	WHO-GHED
Health expenditure per capita, PPP (constant 2020 USD $)	2015-19	WHO-GHED
GDP per capita, PPP (constant 2020 USD $)	2015-19	WHO-GHED
**Output variables**		
**Health outcomes**		
Life expectancy at birth (years)	2015-19	IHME-GBD
Healthy life expectancy at birth (years)	2015-19	IHME-GBD
Neonatal mortality rate per 1,000 live births *	2015-19	WHO-GHO
Under-5 mortality rate per 1,000 live births *	2015-19	WHO-GHO
DALYs for all causes per 100,000 population *	2015-19	IHME-GBD
DALYs for NCDs per 100,000 population *	2015-19	IHME-GBD
DALYs for maternal causes per 100,000 population *	2015-19	IHME-GBD
DALYs for neonatal causes per 100,000 population *	2015-19	IHME-GBD
**Service coverage**		
UHC service coverage index	2017, 2019	WHO-GHO
UHC index on service capacity on access	2017, 2019	WHO-GHO
UHC index on non-communicable diseases	2017, 2019	WHO-GHO
UHC index on reproductive, maternal, newborn, and child health	2017, 2019	WHO-GHO
UHC index on infectious diseases	2017, 2019	WHO-GHO
**Access to services**		
Skilled birth attendance (%)	2015-19	WHO-GHO
DPT immunization rate (% of children aged 12-23 months)	2015-19	WHO-GHO
**Equity**		
Skilled birth attendance ratio poorest/richest wealth quintiles	Recent	WHO-GHO
Skilled birth attendance ratio rural/urban areas	Recent	WHO-GHO
**Explanatory variables**		
Out-of-pocket as a % of total expenditure	2015-19	WHO-GHED
Hospital beds per 1,000 people	2015-19	WHO-GHO
Average governance quality (1)	2015-19	WB-WGI

Source: Prepared by authors.

Notes: The explanatory variables correspond to variables used in regression on potential determinants. We included indicators from the Institute of Health Metrics and Evaluation (IHME) Global Burden of Disease (GBD), the World Health Organization (WHO) Global Health Expenditure Database (GHED), the WHO Global Health Observatory (GHO), and the World Bank (WB) Worldwide Governance Indicators (WGI) project. (*) For these indicators, we use the inverse of the variable as we want to reflect a "more is better". (1) The "average governance quality" corresponds to the average of sex governance quality indicators: control of corruption, government effectiveness, political stability and absence of violence/terrorism, regulatory and rule of law.
