# Supplementary material for: Efficiency of health systems in middle-income countries and determinants of efficiency in Latin America and the Caribbean
Source: PLoS One. 2024 Sep 5;19(9):e0309772. doi: 10.1371/journal.pone.0309772 (PMC11376550; doi:10.1371/journal.pone.0309772)
Supplement: S2 Table — (PDF) [file pone.0309772.s006.pdf]

S2 Table. Sample averages by country, 2015-2019

Country	Life expectancy at birth	HALE at birth	Under-5 mortality rate	Neonatal mortality rate	DALYs lost per 100,000 people				UHC services coverage index					Births attended by skilled health staff	DPT immunization on DPT (%)	Ratio skilled birth attendance		Out-of-pocket as a % of CHE	Hospital beds per 1,000 people	Average governance quality
					All causes	*NCDs*	*Maternal causes*	*Neonatal causes*	*Total*	*Service capacity*	*NCDs*	*RMNC health*	*Infectious diseases*			*Poor / Rich*	*Rural / Urban*			
ARG	76.42	66.70	10.29	6.10	27,868	21,307	61	862	73.70	85.72	48.80	88.33	79.86	98.30	88.20	0.996		27.21	4.97	-0.07
BHS	73.60	64.47	15.29	7.17	31,089	22,429	65	793	68.51	63.84	52.39	84.15	78.34	98.75	92.40			27.32	2.90	0.65
BLZ	74.38	65.01	13.74	9.12	25,865	16,030	101	1,637	65.41	53.18	65.97	80.02	65.25	95.14	94.20	0.936	0.977	23.58	1.13	-0.27
BOL	71.66	62.81	28.70	15.28	31,334	18,671	302	3,425	65.60	62.76	73.84	70.17	57.31	86.57	83.60	0.346	0.550	25.31	1.23	-0.64
BRA	75.41	64.76	15.64	9.37	30,440	21,316	66	1,657	75.16	95.46	55.36	76.24	79.28	98.68	86.20	0.744	0.806	24.66	2.12	-0.20
BRB	76.45	66.94	13.34	8.86	32,260	26,692	38	1,001	73.72	80.63	57.66	85.91	73.97	98.78	93.80	1.000	1.017	45.30	5.92	0.90
CHL	79.96	69.11	7.44	4.84	24,529	20,018	25	492	78.28	90.72	56.82	90.09	80.90	99.76	95.00			33.72	2.11	0.94
COL	79.96	69.39	14.57	7.93	24,069	16,741	77	1,184	77.18	85.76	71.98	81.82	70.27	98.28	92.00	0.888	0.898	15.14	1.68	-0.19
CRI	79.96	69.31	8.65	6.04	22,776	17,693	33	689	76.26	73.53	68.07	87.32	77.45	97.00	94.80	1.013	0.997	22.16	1.13	0.61
DOM	72.53	63.72	34.81	24.27	31,230	19,726	158	3,422	64.06	59.08	52.06	83.74	65.41	99.62	86.40	0.973	0.990	28.92	1.52	-0.21
ECU	76.07	66.48	14.38	6.99	25,087	16,941	110	1,442	78.28	83.99	76.28	80.79	72.73	95.39	83.20	0.424	0.632	32.65	1.48	-0.47
GTM	72.20	62.60	26.38	12.42	29,063	16,854	174	1,760	56.73	32.98	67.68	70.27	66.21	69.70	82.80	0.413	0.681	55.89	0.43	-0.62
GUY	66.87	58.02	31.19	19.04	40,501	25,784	204	2,721	73.29	79.75	58.98	82.05	74.79	95.75	96.60	0.953	0.997	32.20	1.72	-0.24
HND	71.43	62.54	18.00	9.79	26,896	17,478	165	1,880	61.27	37.41	68.87	80.01	68.50	79.78	92.40	0.830	0.920	50.91	0.65	-0.64
HTI	63.00	54.75	66.01	26.18	49,856	23,319	794	4,666	45.83	25.47	63.82	50.71	53.67	41.60	61.40	0.186	0.498	37.63		-1.19
JAM	76.25	66.56	14.61	10.32	26,672	20,644	67	1,746	69.00	75.21	54.35	86.32	64.27	99.70	95.20	0.965	0.983	17.37	1.73	0.20
MEX	75.56	65.33	15.17	8.51	26,752	19,748	61	1,216	73.24	77.44	64.80	82.57	69.48	97.43	87.00	0.920	0.943	41.76	0.99	-0.36
NIC	75.06	65.53	17.97	10.85	22,494	16,294	63	1,180	68.62	68.60	67.74	76.05	62.82	94.07	98.00	0.635	0.766	34.14	0.93	-0.73
PAN	79.65	68.97	15.92	8.87	23,295	16,426	89	1,120	75.43	89.89	66.22	81.31	66.93	94.60	83.20	0.721	0.786	33.98	2.27	0.14
PER	79.82	69.58	14.45	7.49	22,322	14,792	101	1,626	77.38	77.51	82.69	76.42	73.37	93.14	86.80	0.776	0.816	29.12	1.58	-0.13
PRY	76.40	66.30	20.90	11.17	24,425	17,188	105	1,030	60.06	60.31	36.74	84.82	69.39	96.70	89.80	0.879	0.920	43.27	0.81	-0.40
SLV	75.07	65.00	14.36	6.98	28,950	18,876	53	989	74.13	75.79	71.39	81.52	68.49	99.90	86.20	0.944	0.966	36.08	1.09	-0.29
SUR	72.53	62.78	19.23	11.97	33,699	23,194	128	2,571	67.22	71.02	55.91	72.59	70.94	94.20	75.00	0.972	0.988	20.81	3.02	-0.17
TTO	74.85	65.20	18.26	11.68	32,583	25,437	39	1,127	72.44	78.21	53.83	81.14	80.66	100.00	94.80	1.004	0.989	43.41	3.02	0.09
URY	77.35	67.38	7.82	4.44	30,751	24,639	28	580	78.57	92.79	52.69	92.64	84.18	99.98	93.60	1.011	0.984	16.31	2.45	0.88
VEN	75.46	65.73	23.20	14.60	28,579	19,152	134	1,525	69.68	69.21	64.02	77.58	68.70	99.10	72.20			29.58	0.82	-1.61
**LAC**	**74.92**	**65.19**	**19.24**	**10.78**	**28,976**	**19,900**	**125**	**1,628**	**69.96**	**71.01**	**61.88**	**80.18**	**70.89**	**93.15**	**87.88**	**0.806**	**0.868**	**31.86**	**1.91**	**-0.15**
**MICS**	**70.80**	**61.87**	**29.94**	**14.57**	**34,649**	**21,013**	**189**	**2,507**	**60.71**	**60.35**	**56.07**	**71.69**	**61.70**	**89.60**	**87.86**	**0.750**	**0.846**	**35.94**	**2.36**	**-0.32**
**OECD**	**80.78**	**69.64**	**4.81**	**2.77**	**28,501**	**24,258**	**13**	**373**	**81.01**	**92.37**	**62.73**	**89.55**	**83.72**	**98.75**	**94.94**	**0.937**	**0.947**	**20.18**	**4.49**	**1.07**
**Total**	**73.47**	**63.96**	**23.15**	**11.42**	**33,096**	**22,005**	**141**	**1,918**	**66.23**	**69.11**	**57.63**	**76.66**	**67.81**	**92.01**	**89.63**	**0.759**	**0.850**	**31.86**	**3.35**	**0.07**

Source: Author's calculations.

Notes: The data for health outcomes, access to services and the explanatory variables are averages between 2015-2019; the service coverage is an average between 2017 and 2019 and the equity measures use the most recent data available.
