# Supplementary material for: Efficiency of health systems in middle-income countries and determinants of efficiency in Latin America and the Caribbean
Source: PLoS One. 2024 Sep 5;19(9):e0309772. doi: 10.1371/journal.pone.0309772 (PMC11376550; doi:10.1371/journal.pone.0309772)
Supplement: S3 Table — (PDF) [file pone.0309772.s007.pdf]

S3 Table. Efficiency score by output indicator, 2015-2019

Country	Life expectancy at birth	HALE at birth	Under-5 mortality rate	Neonatal mortality rate	DALYs lost per 100,000 people				UHC services coverage index					Births attended by skilled health staff	DPT immunization DPT (%)	Ratio skilled birth attendance	
					All causes	*NCDs*	*Maternal causes*	*Neonatal causes*	*Total*	*Service capacity*	*NCDs*	*RMNC health*	*Infectious diseases*			*Poor / Rich*	*Rural / Urban*
ARG	0.938	0.940	0.993	0.995	0.917	0.937	0.999	0.995	0.896	0.865	0.603	0.953	0.935	0.983	0.891	0.983	
BHS	0.910	0.915	0.988	0.994	0.860	0.901	0.999	0.995	0.837	0.640	0.634	0.924	0.939	0.988	0.933		
BLZ	0.974	0.978	0.997	0.996	0.945	0.969	0.999	0.992	0.899	0.596	0.842	0.950	0.828	0.951	0.952	0.935	0.964
BOL	0.931	0.937	0.982	0.989	0.868	0.940	0.997	0.972	0.887	0.695	0.934	0.826	0.723	0.866	0.844	0.345	0.543
BRA	0.941	0.927	0.990	0.993	0.889	0.925	0.999	0.988	0.941	0.975	0.671	0.860	0.978	0.987	0.871	0.741	0.793
BRB	0.967	0.968	0.993	0.994	0.921	0.930	1.000	0.994	0.945	0.851	0.743	0.980	0.960	0.989	0.948	0.999	1.000
CHL	0.981	0.974	0.996	0.996	0.965	0.952	1.000	0.998	0.946	0.912	0.705	0.973	0.941	0.998	0.960		
COL	0.996	0.992	0.991	0.994	0.952	0.973	0.999	0.992	0.965	0.875	0.870	0.920	0.851	0.983	0.929	0.880	0.883
CRI	0.986	0.982	0.995	0.995	0.972	0.968	1.000	0.996	0.933	0.744	0.829	0.951	0.914	0.970	0.958	1.000	0.979
DOM	0.907	0.915	0.971	0.979	0.866	0.930	0.999	0.970	0.802	0.603	0.630	0.945	0.814	0.996	0.873	0.970	0.974
ECU	0.960	0.963	0.993	0.996	0.947	0.970	0.999	0.990	1.000	0.887	0.931	0.927	0.899	0.954	0.840	0.422	0.622
GTM	0.944	0.942	0.987	0.994	0.929	0.959	0.999	0.993	0.793	0.367	0.868	0.832	0.872	0.697	0.837	0.413	0.675
GUY	0.848	0.845	0.977	0.985	0.751	0.859	0.998	0.978	0.954	0.836	0.725	0.945	0.942	0.958	0.976	0.951	0.983
HND	0.953	0.960	0.998	0.997	0.968	0.951	0.999	0.994	0.870	0.431	0.897	0.994	0.921	0.799	0.939	0.836	0.911
HTI	1.000	1.000	1.000	1.000	1.000	1.000	1.000	1.000	1.000	0.784	1.000	1.000	1.000	0.678	0.815	0.499	0.889
JAM	0.976	0.977	0.995	0.994	0.931	0.925	1.000	0.988	0.908	0.811	0.674	1.000	0.806	0.997	0.962	0.962	0.969
MEX	0.943	0.935	0.990	0.994	0.928	0.935	0.999	0.992	0.912	0.787	0.784	0.920	0.856	0.974	0.879	0.916	0.926
NIC	0.999	1.000	0.995	0.995	1.000	0.977	1.000	0.998	0.972	0.788	0.882	0.940	0.830	0.942	0.995	0.638	0.758
PAN	0.972	0.968	0.987	0.992	0.947	0.972	0.999	0.991	0.901	0.900	0.801	0.878	0.777	0.946	0.840	0.711	0.772
PER	1.000	1.000	0.993	1.000	1.000	0.993	0.999	0.988	0.990	0.809	1.000	0.875	0.926	0.931	0.877	0.775	0.804
PRY	0.956	0.952	0.986	0.992	0.947	0.958	0.999	0.994	0.761	0.623	0.444	0.967	0.865	0.967	0.907	0.876	0.906
SLV	0.977	0.969	0.996	0.997	0.928	0.970	1.000	0.997	1.000	0.840	0.914	0.981	0.869	1.000	0.871	0.943	0.953
SUR	0.900	0.894	0.984	0.989	0.822	0.890	0.999	0.978	0.831	0.722	0.676	0.802	0.852	0.942	0.758	0.964	0.971
TTO	0.927	0.927	0.985	0.990	0.860	0.878	1.000	0.992	0.888	0.787	0.656	0.893	0.973	1.000	0.958	0.996	0.971
URY	0.952	0.953	0.995	0.997	0.918	0.926	1.000	0.997	0.962	0.942	0.680	1.000	0.990	1.000	0.945	1.000	0.966
VEN	0.944	0.944	0.983	0.989	0.905	0.940	0.999	0.989	0.873	0.707	0.774	0.876	0.858	0.991	0.729		
**Including all countries**																	
**LAC**	0.953	0.952	0.990	0.993	0.920	0.944	0.999	0.991	0.910	0.761	0.776	0.927	0.889	0.942	0.896	0.815	0.873
**MICS**	0.932	0.936	0.985	0.993	0.880	0.937	0.999	0.987	0.865	0.691	0.728	0.884	0.834	0.927	0.901	0.801	0.868
**OECD**	0.972	0.964	0.998	0.998	0.938	0.938	1.000	0.998	0.950	0.936	0.814	0.970	0.939	0.988	0.960	0.929	0.931
**Total**	0.942	0.943	0.989	0.994	0.894	0.937	0.999	0.990	0.888	0.758	0.749	0.908	0.864	0.943	0.916	0.807	0.871
**Without Haiti**																	
**LAC**	0.951	0.950	0.990	0.993	0.917	0.941	0.999	0.990	0.907	0.760	0.767	0.925	0.885	0.952	0.899	0.830	0.873
**MICS**	0.933	0.937	0.986	0.993	0.881	0.939	0.999	0.988	0.870	0.693	0.732	0.883	0.838	0.930	0.902	0.805	0.868
**OECD**	0.972	0.964	0.998	0.998	0.938	0.938	1.000	0.998	0.950	0.936	0.814	0.970	0.939	0.988	0.960	0.929	0.931
**Total**	0.943	0.943	0.989	0.994	0.895	0.938	0.999	0.991	0.891	0.759	0.751	0.907	0.867	0.945	0.916	0.810	0.870

Source: Author’s calculations.

Notes: Average efficiency scores for MICS and OECD countries include countries in LAC. Total corresponds to the enlarged sample with LAC, MICS and OECD. Results from output-oriented DEA model using as input variables public health spending per capita, GDP per capita, and population aged 65 and above. Results without Haiti corresponds to re-running all the main analysis described in section “Methods and data” excluding Haiti.
