# Supplementary material for: Efficiency of health systems in middle-income countries and determinants of efficiency in Latin America and the Caribbean
Source: PLoS One. 2024 Sep 5;19(9):e0309772. doi: 10.1371/journal.pone.0309772 (PMC11376550; doi:10.1371/journal.pone.0309772)
Supplement: S4 Table — (PDF) [file pone.0309772.s008.pdf]

S4 Table. Efficiency score by output indicator, 2010-2014

Country	Life expectancy at birth	HALE at birth	Under-5 mortality rate	Neonatal mortality rate	DALYs lost per 100,000 people				UHC services coverage index					Births attended by skilled health staff	DPT immunization DPT (%)	Ratio skilled birth attendance	
					All causes	*NCDs*	*Maternal causes*	*Neonatal causes*	*Total*	*Service capacity*	*NCDs*	*RMNC health*	*Infectious diseases*			*Poor / Rich*	*Rural / Urban*
ARG	0.942	0.944	0.991	0.994	0.917	0.945	0.999	0.992	0.936	0.862	0.661	0.957	0.912	0.974	0.937		
BHS	0.918	0.920	0.990	0.994	0.874	0.917	0.999	0.994	0.830	0.596	0.673	0.935	0.819	0.989	0.986		
BLZ	0.959	0.958	0.994	0.995	0.956	0.980	0.999	0.990	0.914	0.582	0.887	0.956	0.858	0.940	0.968	0.910	0.958
BOL	0.917	0.918	0.974	0.985	0.859	0.946	0.997	0.968	0.814	0.746	0.960	0.799	0.470	0.836	0.911	0.345	0.548
BRA	0.941	0.923	0.989	0.992	0.872	0.927	0.999	0.983	0.991	0.968	0.671	0.874	1.000	0.987	0.976	0.739	0.793
BRB	0.966	0.967	0.991	0.993	0.918	0.928	1.000	0.993	0.992	0.831	0.772	0.918	0.883	0.993	0.907	0.993	1.000
CHL	0.985	0.978	0.996	0.996	0.968	0.959	1.000	0.997	0.947	0.907	0.674	0.957	0.906	0.998	0.933		
COL	1.000	0.991	0.989	0.994	0.948	0.982	0.999	0.989	0.939	0.831	0.886	0.894	0.673	0.981	0.899	0.850	0.870
CRI	1.000	0.995	0.995	0.995	0.988	0.984	1.000	0.995	0.956	0.701	0.818	0.926	0.928	0.982	0.909	0.963	0.962
DOM	0.928	0.929	0.973	0.979	0.885	0.952	0.999	0.966	0.825	0.578	0.667	0.939	0.728	0.987	0.871	0.988	0.970
ECU	0.957	0.953	0.991	0.995	0.936	0.970	0.999	0.987	1.000	0.871	0.965	0.918	0.756	0.914	0.881	0.422	0.621
GTM	0.923	0.914	0.980	0.991	0.910	0.966	0.998	0.989	0.854	0.471	0.898	0.872	0.855	0.613	0.905	0.413	0.680
GUY	0.849	0.841	0.974	0.983	0.765	0.871	0.998	0.977	0.979	0.806	0.755	0.878	0.979	0.938	0.972	0.792	0.895
HND	0.917	0.919	0.992	0.994	0.922	0.954	0.998	0.989	0.828	0.401	0.917	0.975	0.847	0.830	0.997	0.628	0.801
HTI	0.736	0.736	0.915	0.982	0.249	0.913	0.992	0.968	0.626	0.384	1.000	0.708	0.511	0.439	0.692	0.331	0.700
JAM	0.982	0.977	0.993	0.992	0.962	0.945	0.999	0.987	0.924	0.778	0.712	0.996	0.763	0.987	0.943	0.962	0.970
MEX	0.956	0.944	0.988	0.994	0.935	0.953	0.999	0.989	0.937	0.800	0.761	0.938	0.797	0.962	0.931		
NIC	0.972	0.972	0.991	0.993	1.000	0.978	1.000	0.997	0.916	0.743	0.898	0.989	0.684	0.903	0.996	0.641	0.770
PAN	0.977	0.973	0.985	0.991	0.939	0.977	0.999	0.991	0.920	0.867	0.821	0.922	0.714	0.932	0.861	0.713	0.773
PER	0.998	0.993	0.991	0.995	0.992	1.000	0.999	0.986	1.000	0.748	1.000	0.893	0.846	0.869	0.919	0.636	0.718
PRY	0.971	0.960	0.984	0.991	0.969	0.970	0.999	0.991	0.765	0.592	0.447	0.980	0.745	0.961	0.925	0.662	0.751
SLV	0.964	0.951	0.993	0.996	0.920	0.976	1.000	0.995	0.982	0.662	0.934	0.972	0.934	0.989	0.922	0.942	0.960
SUR	0.927	0.914	0.986	0.990	0.864	0.916	0.999	0.975	0.870	0.708	0.697	0.847	0.776	0.900	0.802	0.870	0.876
TTO	0.940	0.934	0.984	0.988	0.866	0.894	1.000	0.991	0.908	0.760	0.702	0.846	0.924	0.995	0.921	0.996	0.972
URY	0.960	0.957	0.995	0.997	0.924	0.946	1.000	0.996	1.000	0.955	0.708	1.000	0.898	0.995	0.958	1.000	0.971
VEN	1.000	1.000	1.000	1.000	1.000	1.000	1.000	1.000	1.000	1.000	1.000	1.000	1.000	1.000	1.000		
**LAC**	**0.945**	**0.941**	**0.985**	**0.992**	**0.898**	**0.952**	**0.999**	**0.988**	**0.910**	**0.736**	**0.803**	**0.919**	**0.816**	**0.919**	**0.920**	**0.752**	**0.836**
**MICS**	**0.900**	**0.901**	**0.975**	**0.990**	**0.823**	**0.939**	**0.998**	**0.979**	**0.786**	**0.637**	**0.756**	**0.846**	**0.642**	**0.874**	**0.909**	**0.755**	**0.840**
**OECD**	**0.976**	**0.967**	**0.998**	**0.998**	**0.938**	**0.939**	**1.000**	**0.998**	**0.958**	**0.932**	**0.810**	**0.960**	**0.887**	**0.989**	**0.965**	**0.911**	**0.922**
**Total**	**0.921**	**0.919**	**0.981**	**0.992**	**0.854**	**0.938**	**0.999**	**0.984**	**0..834**	**0.719**	**0.770**	**0.878**	**0.714**	**0.904**	**0.924**	**0.764**	**0.845**

Source: Author's calculations.

Notes: Average efficiency scores for MICS and OECD countries include countries in LAC. Total corresponds to the enlarged sample with LAC, MICS, and OECD. Results from output-oriented DEA model using as input variables public health spending per capita, GDP per capita, and population aged 65 and above.
