# Supplementary material for: Efficiency of health systems in middle-income countries and determinants of efficiency in Latin America and the Caribbean
Source: PLoS One. 2024 Sep 5;19(9):e0309772. doi: 10.1371/journal.pone.0309772 (PMC11376550; doi:10.1371/journal.pone.0309772)
Supplement: S5 Table — (PDF) [file pone.0309772.s009.pdf]

S5 Table. Potential gains due to efficient health spending by output indicator, 2015-2019

Country	Life expectancy at birth	HALE at birth	Under-5 mortality rate	Neonatal mortality rate	DALYs lost per 100,000 people				UHC services coverage index					Births attended by skilled health staff	DPT immunization DPT (%)	Ratio skilled birth attendance	
					All causes	*NCDs*	*Maternal causes*	*Neonatal causes*	*Total*	*Service capacity*	*NCDs*	*RMNC health*	*Infectious diseases*			*Poor / Rich*	*Rural / Urban*
ARG	4.72	3.99	7.14	4.82	5,967	4,974	55	544	7.69	11.60	19.38	4.11	5.22	1.67	9.62	0.02	
BHS	6.61	5.48	11.71	5.81	9,620	7,691	58	459	11.16	23.00	19.20	6.38	4.76	1.23	6.16		
BLZ	1.92	1.41	2.53	4.23	4,110	2,642	71	797	6.64	21.47	10.41	3.98	11.20	4.62	4.53	0.06	0.04
BOL	4.94	3.97	17.90	10.59	9,048	4,879	275	2,658	7.41	19.17	4.90	12.18	15.89	11.63	13.00	0.23	0.25
BRA	4.44	4.70	9.55	6.78	7,695	5,930	55	1,228	4.40	2.38	18.21	10.71	1.76	1.30	11.15	0.19	0.17
BRB	2.50	2.17	7.27	5.67	5,365	5,160	25	563	4.04	12.05	14.84	1.69	2.95	1.13	4.86	0.00	0.00
CHL	1.53	1.81	4.40	3.58	2,644	3,815	19	196	4.22	7.97	16.77	2.39	4.75	0.24	3.84		
COL	0.29	0.55	9.18	5.54	3,673	2,215	70	778	2.70	10.69	9.32	6.56	10.49	1.69	6.51	0.11	0.10
CRI	1.09	1.27	5.42	4.73	2,129	2,607	27	355	5.09	18.84	11.65	4.30	6.67	2.91	4.02	0.00	0.02
DOM	6.72	5.44	28.00	20.68	9,213	5,602	145	2,881	12.66	23.44	19.28	4.63	12.14	0.38	11.00	0.03	0.03
ECU	3.06	2.48	6.42	3.52	3,967	2,517	95	973	0.00	9.51	5.26	5.91	7.32	4.40	13.28	0.25	0.24
GTM	4.02	3.60	13.12	6.32	5,022	3,401	145	715	11.72	20.88	8.95	11.77	8.48	21.12	13.46	0.24	0.22
GUY	10.19	9.02	22.69	14.77	14,832	10,496	187	2,108	3.40	13.05	16.24	4.50	4.33	4.07	2.34	0.05	0.02
HND	3.39	2.51	2.20	3.04	2,351	4,034	128	561	7.98	21.29	7.11	0.47	5.38	16.03	5.62	0.14	0.08
HTI	0.00	0.00	0.00	0.00	0.00	0.00	0.00	0.00	0.00	5.50	0.00	0.00	0.00	13.40	11.37	0.09	0.06
JAM	1.85	1.52	5.37	6.27	5,081	5,939	47	1,173	6.33	14.18	17.71	0.00	12.44	0.30	3.65	0.04	0.03
MEX	4.34	4.25	9.98	6.01	5,294	5,230	50	792	6.47	16.50	14.02	6.57	10.00	2.50	10.55	0.08	0.07
NIC	0.09	0.00	4.60	5.10	0.00	1,967	26	228	1.92	14.54	8.00	4.56	10.66	5.41	0.45	0.23	0.19
PAN	2.20	2.22	12.82	7.59	4,055	2,343	83	849	7.43	9.00	13.19	9.95	14.94	5.11	13.28	0.21	0.18
PER	0.00	0.00	6.87	3.80	233	0.00	86	1,137	0.77	14.84	0.00	9.52	5.41	6.39	10.70	0.17	0.16
PRY	3.33	3.17	13.66	7.63	4,010	3,444	92	566	14.36	22.72	20.42	2.77	9.40	3.19	8.35	0.11	0.09
SLV	1.73	2.00	4.15	2.50	5,115	2,452	27	321	0.00	12.10	6.12	1.55	8.97	0.04	11.13	0.05	0.05
SUR	7.26	6.66	15.35	10.50	11,820	8,438	120	2,163	11.34	19.77	18.11	14.40	10.51	5.46	18.18	0.04	0.03
TTO	5.45	4.77	14.53	10.24	9,419	9,100	31	776	8.08	16.66	18.52	8.67	2.18	0.00	4.02	0.00	0.03
URY	3.71	3.20	4.69	3.17	5,689	5,564	22	264	2.98	5.40	16.87	0.00	0.86	0.02	5.11	0.00	0.03
VEN	4.21	3.71	16.90	11.31	6,806	4,859	121	1,060	8.85	20.30	14.45	9.64	9.76	0.89	19.55		
**Including all countries**																	
**LAC**	3.45	3.07	9.86	6.70	5,506	4,435	79	929	6.06	14.88	12.65	5.66	7.56	4.43	8.68	0.10	0.09
**MICS**	4.58	3.79	13.75	6.93	6,854	4,690	108	1,206	7.36	13.69	13.65	7.29	8.68	4.99	7.34	0.10	0.09
**OECD**	2.25	2.51	2.03	1.59	4,248	4,613	8	162	3.91	5.59	10.86	2.62	4.71	1.20	3.73	0.06	0.06
**Total**	4.00	3.48	10.65	5.56	6,238	4,738	81	916	6.50	11.54	13.03	6.00	7.53	3.97	6.48	0.09	0.08
**Without Haiti**																	
**LAC**	3.58	3.20	10.26	6.97	5,727	4,612	83	966	6.31	15.25	13.16	5.89	7.86	4.07	8.57	0.10	0.10
**MICS**	4.50	3.74	13.02	6.77	6,741	4,552	106	1,120	7.16	13.60	13.47	7.32	8.51	4.89	7.31	0.010	0.09
**OECD**	2.25	2.51	2.03	1.59	4,248	4,613	8	162	3.91	5.59	10.86	2.62	4.71	1.20	3.73	0.06	0.06
**Total**	3.95	3.44	10.12	5.44	6,161	4,638	80	857	6.36	11.48	12.90	6.02	7.41	3.89	6.46	0.09	0.08

Source: Author's calculations.

Notes: Average potential gains for MICS and OECD countries include countries in LAC. Total corresponds to the enlarged sample with LAC, MICS and OECD. Results from output-oriented DEA model using as input variables public health spending per capita, GDP per capita, and population aged 65 and above. Results without Haiti corresponds to re-running all the main analysis described in section "Methods and data" excluding Haiti.
