# Supplementary material for: Efficiency of health systems in middle-income countries and determinants of efficiency in Latin America and the Caribbean
Source: PLoS One. 2024 Sep 5;19(9):e0309772. doi: 10.1371/journal.pone.0309772 (PMC11376550; doi:10.1371/journal.pone.0309772)
Supplement: S6 Table — (PDF) [file pone.0309772.s010.pdf]

S6 Table. Potential gains due to efficient health spending by output indicator (percent relative to baseline), 2015-2019

Country	Life expectancy at birth	HALE at birth	Under-5 mortality rate	Neonatal mortality rate	DALYs lost per 100,000 people				UHC services coverage index					Births attended by skilled health staff	DPT immunization DPT (%)	Ratio skilled birth attendance	
					All causes	*NCDs*	*Maternal causes*	*Neonatal causes*	*Total*	*Service capacity*	*NCDs*	*RMNC health*	*Infectious diseases*			*Poor / Rich*	*Rural / Urban*
ARG	6.18	5.98	69.33	79.12	21.41	23.35	90.18	63.13	10.43	13.54	39.71	4.66	6.54	1.70	10.91	1.65	
BHS	8.99	8.51	76.59	81.11	30.94	34.29	89.30	57.85	16.29	36.03	36.64	7.58	6.07	1.25	6.67		
BLZ	2.58	2.17	18.37	46.36	15.89	16.48	70.03	48.66	10.15	40.38	15.78	4.98	17.16	4.86	4.81	6.52	3.60
BOL	6.90	6.32	62.38	69.28	28.88	26.13	90.90	77.61	11.30	30.54	6.64	17.37	27.74	13.43	15.56	65.49	45.74
BRA	5.88	7.25	61.05	72.37	25.28	27.82	83.87	74.10	5.86	2.49	32.90	14.05	2.22	1.32	12.93	25.94	20.69
BRB	3.27	3.24	54.48	64.01	16.63	19.33	67.01	56.23	5.47	14.94	25.74	1.96	3.98	1.15	5.18	0.12	0.00
CHL	1.91	2.62	59.21	74.01	10.78	19.06	76.42	39.91	5.39	8.79	29.51	2.65	5.87	0.24	4.04		
COL	0.36	0.79	63.05	69.93	15.26	13.23	91.05	65.73	3.49	12.47	12.95	8.02	14.93	1.72	7.07	11.97	11.65
CRI	1.36	1.84	62.63	78.31	9.35	14.73	81.54	51.54	6.67	25.62	17.11	4.93	8.61	3.00	4.24	0.00	2.06
DOM	9.26	8.54	80.44	85.20	29.50	28.40	92.10	84.19	19.77	39.68	37.04	5.53	18.57	0.38	12.73	3.02	2.62
ECU	4.02	3.72	44.63	50.33	15.81	14.86	86.55	67.44	0.00	11.32	6.90	7.31	10.06	4.61	15.96	57.77	37.78
GTM	5.57	5.76	49.74	50.91	17.28	20.18	83.31	40.62	20.67	63.30	13.22	16.76	12.81	30.30	16.25	58.69	32.52
GUY	15.23	15.55	72.76	77.58	36.62	40.71	91.88	77.44	4.63	16.36	27.53	5.49	5.79	4.25	2.42	4.93	1.69
HND	4.74	4.01	12.20	31.03	8.74	23.08	77.54	29.84	13.03	56.91	10.32	0.59	7.85	20.09	6.09	16.43	8.87
HTI	0.00	0.00	0.00	0.00	0.00	0.00	0.00	0.00	0.00	21.59	0.00	0.00	0.00	32.21	18.52	50.08	11.13
JAM	2.43	2.28	36.74	60.76	19.05	28.77	70.08	67.18	9.17	18.85	32.58	0.00	19.36	0.30	3.84	3.77	3.07
MEX	5.74	6.50	65.81	70.59	19.79	26.48	82.07	65.15	8.84	21.31	21.64	7.96	14.39	2.57	12.12	8.41	7.36
NIC	0.12	0.00	25.62	47.06	0.00	12.07	42.31	19.36	2.80	21.20	11.81	6.00	16.97	5.75	0.46	36.23	24.25
PAN	2.76	3.22	80.52	85.52	17.41	14.27	93.68	75.83	9.85	10.01	19.92	12.24	22.33	5.40	15.96	28.87	22.78
PER	0.00	0.00	47.56	50.66	1.04	0.00	85.64	69.92	0.99	19.15	0.00	12.46	7.38	6.86	12.32	22.54	19.65
PRY	4.36	4.78	65.36	68.35	16.42	20.04	87.20	54.99	23.91	37.68	55.57	3.27	13.54	3.30	9.29	12.37	9.39
SLV	2.30	3.07	28.91	35.86	17.67	12.99	51.47	32.40	0.00	15.97	8.57	1.90	13.10	0.04	12.91	5.66	4.67
SUR	10.02	10.61	79.80	87.67	35.07	36.38	94.23	84.12	16.88	27.84	32.39	19.84	14.82	5.80	24.24	3.64	2.93
TTO	7.28	7.31	79.55	87.68	28.91	35.78	80.05	68.91	11.15	21.30	34.40	10.68	2.70	0.00	4.24	0.39	2.87
URY	4.80	4.75	59.93	71.55	18.50	22.58	78.76	45.47	3.79	5.82	32.01	0.00	1.02	0.02	5.45	0.00	3.35
VEN	5.58	5.64	72.83	77.42	23.81	25.37	90.69	69.50	12.71	29.33	22.57	12.42	14.21	0.90	27.07		
**Including all countries**																	
**LAC**	**4.60**	**4.71**	**51.25**	**62.16**	**19.00**	**22.28**	**63.69**	**57.02**	**8.67**	**20.95**	**20.44**	**7.06**	**10.66**	**4.75**	**9.88**	**12.55**	**10.81**
**MICS**	**6.47**	**6.13**	**45.48**	**47.24**	**19.70**	**22.31**	**56.48**	**47.48**	**12.12**	**22.67**	**24.32**	**10.17**	**14.07**	**5.58**	**8.37**	**12.85**	**10.15**
**OECD**	**2.79**	**3.60**	**42.24**	**57.17**	**14.90**	**19.02**	**60.31**	**43.45**	**4.83**	**6.06**	**17.32**	**2.92**	**5.62**	**1.21**	**3.93**	**6.84**	**6.78**
**Total**	**5.45**	**5.44**	**45.77**	**48.52**	**18.81**	**21.51**	**56.89**	**47.39**	**9.81**	**16.68**	**22.59**	**7.82**	**11.11**	**4.32**	**7.23**	**12.43**	**9.96**

Source: Author's calculations.

Notes: Average potential gains for MICS and OECD countries include countries in LAC. Total corresponds to the enlarged sample with LAC, MICS and OECD. Results from output-oriented DEA model using as input variables public health spending per capita, GDP per capita, and population aged 65 and above. Results without Haiti corresponds to re-running all the main analysis described in section "Methods and data" excluding Haiti.
