# Supplementary material for: Efficiency of health systems in middle-income countries and determinants of efficiency in Latin America and the Caribbean
Source: PLoS One. 2024 Sep 5;19(9):e0309772. doi: 10.1371/journal.pone.0309772 (PMC11376550; doi:10.1371/journal.pone.0309772)
Supplement: S8 Table — (PDF) [file pone.0309772.s012.pdf]

S8 Table. Comparison of potential gains due to efficient health spending by model and country for selected output indicators, 2015-2019

	Life expectancy at birth						Neonatal mortality rate						UHC service coverage index						Births attended by skilled health staff					
**Country**	**(1)**	**(2)**	**(3)**	**(4)**	**(5)**	**(6)**	**(1)**	**(2)**	**(3)**	**(4)**	**(5)**	**(6)**	**(1)**	**(2)**	**(3)**	**(4)**	**(5)**	**(6)**	**(1)**	**(2)**	**(3)**	**(4)**	**(5)**	**(6)**
ARG	5.28	5.30	5.27	4.84	4.72	4.79	4.90	4.86	4.89	4.82	4.82	4.82	9.04	9.42	9.43	7.69	7.69	7.69	1.67	1.67	1.67	1.67	1.67	1.67
BHS	7.36	6.61	7.36	7.36	6.61	7.36	5.92	5.81	5.92	5.92	5.81	5.92	12.22	11.16	12.54	12.22	11.16	12.54	1.23	1.23	1.23	1.23	1.23	1.23
BLZ	3.03	3.72	3.59	1.91	1.92	1.92	4.56	5.03	5.05	4.23	4.23	4.23	7.24	8.79	8.81	6.41	6.64	6.64	4.62	4.62	4.62	4.62	4.62	4.62
BOL	6.52	7.20	6.86	4.94	4.94	4.94	11.20	11.63	11.59	10.59	10.59	10.59	9.41	10.83	10.40	7.31	7.41	7.41	11.63	11.63	11.63	11.63	11.63	11.63
BRA	5.08	4.52	5.07	4.48	4.44	4.48	8.00	7.01	8.00	6.82	6.78	6.82	5.86	4.94	6.15	4.40	4.40	4.40	1.30	1.30	1.30	1.30	1.30	1.30
BRB	3.77	3.43	3.45	2.79	2.50	2.68	7.43	6.02	6.32	6.77	5.67	6.21	6.34	5.86	5.93	3.82	4.04	3.96	1.21	1.21	1.21	1.18	1.13	1.15
CHL	2.06	1.87	1.79	1.87	1.53	1.51	3.65	3.59	3.61	3.62	3.58	3.59	5.32	5.34	5.24	4.37	4.22	4.15	0.24	0.24	0.24	0.24	0.24	0.24
COL	0.61	0.94	0.88	0.29	0.29	0.29	6.55	6.57	6.58	5.54	5.54	5.54	3.60	4.48	4.40	2.70	2.70	2.70	1.69	1.69	1.69	1.69	1.69	1.69
CRI	1.12	1.47	1.26	1.05	1.09	1.03	4.74	4.75	4.76	4.73	4.73	4.73	5.35	6.29	5.95	4.99	5.09	5.02	2.91	2.91	2.91	2.91	2.91	2.91
DOM	6.74	6.72	6.74	6.74	6.72	6.74	20.81	20.68	20.96	20.81	20.68	20.96	12.60	12.66	12.73	12.60	12.66	12.73	0.38	0.38	0.38	0.38	0.38	0.38
ECU	3.90	3.87	3.83	3.06	3.06	3.06	4.72	4.44	4.59	3.52	3.52	3.52	1.84	1.96	1.92	0.00	0.00	0.00	4.40	4.40	4.40	4.40	4.40	4.40
GTM	5.57	4.02	4.19	4.42	4.02	4.19	8.13	6.61	7.12	7.75	6.32	6.60	13.93	11.72	12.22	12.94	11.72	12.22	21.12	21.12	21.12	21.12	21.12	21.12
GUY	10.08	10.19	9.98	10.08	10.19	9.98	14.82	14.98	14.96	14.82	14.77	14.75	2.95	3.40	3.13	2.68	3.40	3.13	4.07	4.07	4.07	4.07	4.07	4.07
HND	5.31	4.57	4.59	3.39	3.39	3.39	5.05	3.68	3.82	3.85	3.04	3.11	9.61	8.53	8.84	7.98	7.98	7.98	16.13	16.13	16.13	16.03	16.03	16.03
HTI	9.63	0.00	10.01	5.89	0.00	5.89	17.82	0.00	14.65	15.93	0.00	13.62	7.85	0.00	13.79	7.64	0.00	12.62	22.20	14.51	23.78	22.07	13.40	23.41
JAM	2.98	3.37	3.48	1.85	1.85	1.85	6.56	6.81	7.24	6.30	6.27	6.30	7.82	8.60	9.35	6.03	6.33	6.55	0.30	0.30	0.30	0.30	0.30	0.30
MEX	4.60	4.34	4.34	4.60	4.34	4.34	7.12	6.01	6.32	7.12	6.01	6.32	6.80	6.47	6.52	6.80	6.47	6.52	2.50	2.50	2.50	2.50	2.50	2.50
NIC	2.77	3.02	2.70	0.09	0.09	0.09	6.44	6.69	6.62	5.10	5.10	5.10	5.38	6.12	5.78	1.92	1.92	1.92	5.58	5.58	5.58	5.41	5.41	5.41
PAN	2.45	2.25	2.15	2.38	2.20	2.09	7.65	7.59	7.61	7.64	7.59	7.60	7.84	7.86	7.72	7.50	7.43	7.39	5.11	5.11	5.11	5.11	5.11	5.11
PER	0.00	0.00	0.00	0.00	0.00	0.00	3.98	4.03	4.20	3.98	3.80	3.92	1.51	1.50	1.80	0.40	0.77	0.84	6.39	6.39	6.39	6.39	6.39	6.39
PRY	3.62	3.33	3.39	3.39	3.33	3.37	8.94	7.81	8.20	8.17	7.63	7.93	15.09	14.82	14.92	14.52	14.36	14.51	3.19	3.19	3.19	3.19	3.19	3.19
SLV	4.59	4.52	4.50	1.73	1.73	1.73	3.94	3.66	3.78	2.50	2.50	2.50	5.21	5.13	5.13	0.00	0.00	0.00	0.10	0.10	0.10	0.04	0.04	0.04
SUR	7.24	7.44	7.38	7.22	7.26	7.26	10.53	10.54	10.54	10.50	10.50	10.50	11.43	11.94	11.88	11.28	11.34	11.34	5.46	5.46	5.46	5.46	5.46	5.46
TTO	5.87	5.45	5.44	5.87	5.45	5.44	10.31	10.24	10.25	10.30	10.24	10.25	8.64	8.08	8.11	8.61	8.08	8.11	0.00	0.00	0.00	0.00	0.00	0.00
URY	4.20	4.34	4.43	4.00	3.71	3.94	3.22	3.20	3.24	3.17	3.17	3.17	4.54	5.21	5.47	2.98	2.98	2.98	0.02	0.02	0.02	0.02	0.02	0.02
VEN	4.38	4.21	4.38	4.38	4.21	4.38	12.00	11.31	12.10	12.00	11.31	12.10	9.03	8.85	9.19	9.03	8.85	9.19	0.89	0.89	0.89	0.89	0.89	0.89
**LAC**	4.57	4.10	4.50	3.79	3.45	3.72	8.04	7.06	7.80	7.56	6.70	7.33	7.56	7.31	7.97	6.42	6.06	6.64	4.78	4.49	4.84	4.76	4.43	4.81
**MICS**	6.06	6.04	6.20	4.72	4.58	4.67	9.71	8.05	8.66	8.30	6.93	7.27	8.83	9.27	9.79	7.39	7.36	7.64	5.79	5.64	5.98	5.51	4.99	5.30
**OECD**	2.47	2.51	2.45	2.23	2.25	2.21	1.73	1.69	1.71	1.66	1.59	1.61	4.91	5.27	5.09	3.75	3.91	3.67	1.21	1.21	1.21	1.20	1.20	1.20
**Total**	5.15	5.12	5.23	4.12	4.00	4.06	7.59	6.38	6.83	6.56	5.56	5.81	7.86	8.25	8.59	6.49	6.50	6.64	4.56	4.46	4.70	4.35	3.97	4.20

Source: Author's calculations.

Notes: Average potential gains for MICS and OECD countries include countries in LAC. Table C5 presents the potential gains for output-oriented DEA models using different input variables. Model (1) use as input the total health expenditure per capita. Model (2) use as input the total public health expenditure per capita. Model (3) use as input the total public and private health expenditure per capita. Model (4) use as input the total health expenditure per capita, GPD per capita, and population aged 65 and above. Model (5) use as input the total public health expenditure per capita, GPD per capita, and population aged 65 and above. Model (6) use as input the total public and private health expenditure per capita, GPD per capita, and population aged 65 and above.
