# Supplementary material for: Efficiency of health systems in middle-income countries and determinants of efficiency in Latin America and the Caribbean
Source: PLoS One. 2024 Sep 5;19(9):e0309772. doi: 10.1371/journal.pone.0309772 (PMC11376550; doi:10.1371/journal.pone.0309772)
Supplement: S9 Table — (PDF) [file pone.0309772.s013.pdf]

S9 Table. Potential determinants of efficiency, 2015-2019

	Life expectancy at birth	HALE at birth	Under-5 mortality rate	Neonatal mortality rate	DALYs lost per 100000				Births attended by skilled health staff	DPT immunizati on DPT (%)	Ratio skilled birth attendance		UHC service coverage index				
					Total	NCDs	Maternal	Neonatal			poor/rich	rural/urban	Total	Service capacity	NCDs	RMNC	Infectious diseases
	(1)	(2)	(3)	(4)	(5)	(6)	(7)	(8)	(9)	(10)	(11)	(12)	(13)	(14)	(15)	(16)	(17)
**Enlarged sample**																	
OOP health expenditure as % of CHE	0.000	0.001	0.000	0.000	0.001	0.000	0.000*	0.000	-0.003	0.010	0.009	0.020	0.001	0.001	0.000	0.002	0.000
	0.000	0.000	0.000	0.000	(0.001)	0.000	0.000	(0.001)	(0.006)	(0.018)	(0.010)	(0.019)	(0.001)	(0.002)	(0.001)	(0.006)	(0.004)
Hospital beds per 1.000 people	-0.002	0.001	0.013*	0.005*	-0.009*	-0.010***	0.005***	0.041*	0.824***	0.078	0.602	1.032*	0.021*	0.163***	-0.018***	0.036	0.039
	(0.003)	(0.003)	(0.007)	(0.002)	(0.005)	(0.002)	(0.002)	(0.024)	(0.278)	(0.131)	(0.400)	(0.607)	(0.011)	(0.044)	(0.006)	(0.105)	(0.046)
Average governance quality	0.033***	0.020**	0.027*	0.010**	0.058***	0.005	0.004**	0.062*	0.777***	0.613	0.217	0.378	0.088***	0.170**	0.036*	0.324	0.246
	(0.011)	(0.010)	(0.015)	(0.005)	(0.020)	(0.007)	(0.002)	(0.036)	(0.314)	(0.856)	(0.430)	(0.635)	(0.033)	(0.069)	(0.018)	(0.458)	(0.214)
Constant	0.960***	0.936***	0.995***	0.994***	0.922***	0.980***	0.995***	1.023***	1.284***	1.253	0.331	0.172	0.852***	0.510***	0.827***	1.134	1.040**
	(0.023)	(0.018)	(0.019)	(0.007)	(0.034)	(0.015)	(0.002)	(0.055)	(0.422)	(0.899)	(0.439)	(0.605)	(0.055)	(0.106)	(0.037)	(0.844)	(0.507)
Observations	105	106	102	107	106	109	100	101	86	104	57	59	102	108	114	99	109
Number of efficient DMUs	15	14	18	13	14	11	20	19	18	16	12	11	18	12	6	21	11
Model degrees of freedom	3	3	3	3	3	3	3	3	3	3	3	3	3	3	3	3	3
Model chi-squared	9.326	5.362	3.972	5.875	9.215	23.726	9.189	3.517	9.15	0.545	2.452	2.968	10.821	17.785	14.588	0.508	1.562
Model significance, p-value	0.025	0.147	0.265	0.118	0.027	0.000	0.027	0.319	0.027	0.909	0.484	0.397	0.013	0	0.002	0.917	0.668
**LAC**																	
OOP health expenditure as % of CHE	0.000	-0.002	0.000	0.000	0.001	0.000	0.000	0.002	-0.004	0.001	0.010	0.000	-0.002	-0.003	0.000	0.001	0.004*
	(0.002)	(0.002)	(0.001)	(0.002)	(0.002)	(0.001)	0.000	(0.004)	(0.009)	(0.002)	(0.082)	(0.007)	(0.002)	(0.003)	(0.004)	(0.002)	(0.002)
Hospital beds per 1.000 people	-0.008	-0.025	0.004	-0.001	-0.015	-0.015	0.000	-0.001	0.054	-0.024	0.296	-0.111	-0.006	0.042	-0.040	-0.017	0.015
	(0.015)	(0.020)	(0.012)	(0.015)	(0.022)	(0.012)	(0.001)	(0.040)	(0.138)	(0.017)	(1.524)	(0.181)	(0.027)	(0.036)	(0.034)	(0.018)	(0.026)
Average governance quality	0.009	0.042	0.005	0.022	0.029	0.020	0.001	0.024	0.058	0.154***	1.105	0.764	0.054	0.013	-0.059	0.049	0.038
	(0.028)	(0.036)	(0.015)	(0.035)	(0.051)	(0.026)	(0.003)	(0.061)	(0.146)	(0.034)	(5.653)	(0.465)	(0.043)	(0.060)	(0.072)	(0.030)	(0.037)
Constant	0.974***	1.096***	0.991***	1.024***	0.957***	1.008***	1.001***	0.986**	1.201**	0.977***	0.685	1.345**	1.007***	0.854***	0.862***	0.950***	0.788***
	(0.065)	(0.095)	(0.041)	(0.135)	(0.089)	(0.061)	(0.011)	(0.403)	(0.484)	(0.070)	(4.625)	(0.628)	(0.111)	(0.113)	(0.133)	(0.067)	(0.083)
Observations	15	18	17	19	20	19	17	18	17	21	14	16	17	20	23	20	20
Number of efficient DMUs	11	8	9	7	6	7	9	8	9	5	9	6	9	6	3	6	6
Model degrees of freedom	3	3	3	3	3	3	3	3	3	3	3	3	3	3	3	3	3
Model chi-squared	0.267	2.816	0.447	0.556	0.658	1.58	0.77	0.194	1.129	20.494	0.123	2.884	2.259	3.751	4.031	3.017	4.042
Model significance, p-value	0.966	0.421	0.93	0.906	0.883	0.664	0.857	0.979	0.77	0.00	0.989	0.41	0.52	0.29	0.258	0.389	0.257
**MICS**																	
OOP health expenditure as % of CHE	0.001	0.001	0.000	0.000	0.001	-0.001	0.000	0.000	-0.002	0.010	0.009	0.020	0.002	0.001	0.000	0.003	0.001
	(0.001)	(0.001)	0.000	0.000	(0.001)	0.000	0.000	(0.002)	(0.006)	(0.021)	(0.011)	(0.020)	(0.001)	(0.002)	(0.001)	(0.021)	(0.004)
Hospital beds per 1.000 people	0.000	0.003	0.010	0.003*	-0.013	-0.014***	0.006	0.048	0.849***	0.078	0.602	1.032*	0.039**	0.144***	-0.026***	0.017	0.046
	(0.005)	(0.006)	(0.007)	(0.002)	(0.009)	(0.003)	(0.004)	(0.068)	(0.302)	(0.140)	(0.487)	(0.594)	(0.018)	(0.037)	(0.006)	(0.141)	(0.057)
Average governance quality	0.006	0.016	0.011	0.002	0.027	-0.007	0.003	0.052	0.766**	0.673	0.217	0.378	0.031	0.034	-0.046*	0.138	0.156
	(0.017)	(0.022)	(0.012)	(0.004)	(0.036)	(0.012)	(0.003)	(0.082)	(0.352)	(1.014)	(0.524)	(0.601)	(0.043)	(0.063)	(0.024)	(0.331)	(0.160)
Constant	0.939***	0.960***	0.992***	0.991***	0.951***	1.002***	0.994***	1.044***	1.290***	1.283	0.331	0.172	0.765***	0.444***	0.799***	1.028	0.947***
	(0.027)	(0.036)	(0.023)	(0.006)	(0.061)	(0.020)	(0.004)	(0.191)	(0.383)	(1.030)	(0.617)	(1.018)	(0.060)	(0.095)	(0.036)	(0.908)	(0.367)
Observations	70	75	75	77	75	77	70	73	65	75	57	59	72	76	83	72	78
Number of efficient DMUs	17	12	12	10	12	10	17	14	16	12	12	11	15	11	4	15	9
Model degrees of freedom	3	3	3	3	3	3	3	3	3	3	3	3	3	3	3	3	3
Model chi-squared	0.768	1.098	2.075	3.917	2.633	24.884	2.344	0.604	8.308	0.547	1.613	3.091	7.337	17.508	20.893	0.289	1.058
Model significance, p-value	0.857	0.778	0.557	0.271	0.452	0.000	0.504	0.896	0.04	0.908	0.657	0.378	0.062	0.001	0	0.962	0.787
**OECD**																	
OOP health expenditure as % of CHE	-0.001	0.000	0.000	0.000	0.000	0.000	0.000	0.000	-0.005	0.001	0.012	0.000	-0.002*	-0.005*	-0.002	0.001	0.000
	(0.001)	(0.001)	0.000	(0.001)	(0.001)	(0.001)	0.000	(0.001)	(0.010)	(0.002)	(0.040)	(0.009)	(0.001)	(0.003)	(0.002)	(0.001)	(0.003)
Hospital beds per 1.000 people	-0.005	-0.003	0.005	0.006	-0.005	-0.004	0.001	0.011	0.157	0.005	0.460	0.006	-0.009*	0.008	-0.017*	0.002	-0.006
	(0.003)	(0.003)	(0.004)	(0.006)	(0.004)	(0.002)	0.000	(0.009)	(0.126)	(0.010)	(0.777)	(0.168)	(0.005)	(0.018)	(0.010)	(0.007)	(0.012)
Average governance quality	0.022**	0.011	0.012*	0.010	0.023	0.004	0.001***	0.020	0.083	0.067***	0.948	0.662	0.037**	0.111**	0.035	0.059***	0.067
	(0.009)	(0.009)	(0.006)	(0.011)	(0.015)	(0.008)	0.000	(0.014)	(0.147)	(0.025)	(2.513)	(0.549)	(0.015)	(0.044)	(0.035)	(0.018)	(0.050)
Constant	0.992***	0.966***	0.994***	0.999***	0.941***	0.954***	0.999***	0.982***	1.336**	0.904***	0.379	1.163*	0.990***	1.024***	0.886***	0.924***	1.008***
	(0.026)	(0.024)	(0.012)	(0.022)	(0.033)	(0.019)	(0.001)	(0.025)	(0.567)	(0.052)	(1.703)	(0.696)	(0.035)	(0.107)	(0.076)	(0.040)	(0.122)

Observations	48	50	48	50	53	52	50	48	38	51	15	17	47	50	55	47	49
Number of efficient DMUs	12	10	12	10	7	8	10	12	13	9	9	6	13	10	5	13	11
Model degrees of freedom	3	3	3	3	3	3	3	3	3	3	3	3	3	3	3	3	3
Model chi-squared	9.765	2.613	4.167	1.411	3.178	2.664	9.215	2.435	1.943	9.807	0.371	1.555	15.055	14.87	4.287	16.008	2.813
Model significance, p-value	0.021	0.455	0.244	0.703	0.365	0.446	0.027	0.487	0.584	0.02	0.946	0.67	0.002	0.002	0.232	0.001	0.421

Source: Author’s calculations.

Notes: Simar-Wilson models estimated with 1,000 bootstrap replications. Robust standard errors in parenthesis. *p<0.1, **p<0.5, **p<0.01.
