# Supplementary material for: Efficiency of health systems in middle-income countries and determinants of efficiency in Latin America and the Caribbean
Source: PLoS One. 2024 Sep 5;19(9):e0309772. doi: 10.1371/journal.pone.0309772 (PMC11376550; doi:10.1371/journal.pone.0309772)
Supplement: S10 Table — (PDF) [file pone.0309772.s014.pdf]

S10 Table. Potential determinants of efficiency with disaggregated analysis by governance quality, 2015-2019

	Life expectancy at birth	HALE at birth	Under-5 mortality rate	Neonatal mortality rate	DALYs lost per 100000				Births attended by skilled health staff	DPT immu nization (%)	Ratio skilled birth attendance		UHC service coverage index				
					Total	NCDs	Maternal	Neonatal			poor/ rich	rural/ urban	Total	Service capacity	NCDs	RMNC	Infectious diseases
	(1)	(2)	(3)	(4)	(5)	(6)	(7)	(8)	(9)	(10)	(11)	(12)	(13)	(14)	(15)	(16)	(17)
**Enlarged sample**																	
OOP health expenditure as % of CHE	0.000	0.001	0.000	0.000	0.001	0.000	0.000	0.000	0.003	0.006	0.010	0.009	0.001	0.000	0.000	0.003	0.001
	0.000	0.000	0.000	0.000	(0.001)	0.000	0.000	0.000	(0.003)	(0.004)	(0.015)	(0.008)	(0.001)	(0.002)	(0.001)	(0.004)	(0.002)
Hospital beds per 1.000 people	-0.002	0.001	0.009***	0.003***	-0.013**	-0.010***	0.002***	0.023**	0.258***	0.028	0.642	0.549*	0.014	0.141***	-0.021***	0.029	0.028
	(0.004)	(0.003)	(0.003)	(0.001)	(0.005)	(0.002)	(0.001)	(0.010)	(0.086)	(0.027)	(0.491)	(0.318)	(0.009)	(0.027)	(0.006)	(0.027)	(0.020)
Average governance quality																	
Voice and Accountability	0.010	0.013	0.002	-0.001	-0.008	-0.007	0.001	0.002	0.186	-0.266	0.296	0.283	-0.002	0.006	-0.002	0.060	-0.054
	(0.014)	(0.013)	(0.005)	(0.002)	(0.022)	(0.010)	(0.001)	(0.012)	(0.120)	(0.234)	(0.452)	(0.273)	(0.033)	(0.057)	(0.026)	(0.099)	(0.077)
Political Stability & Absence Violence	-0.011	-0.008	-0.007	-0.002	-0.005	-0.009	-0.001	0.005	-0.050	0.189*	-0.437	-0.321	-0.031	-0.131**	-0.022	-0.040	0.032
	(0.012)	(0.011)	(0.005)	(0.002)	(0.019)	(0.009)	(0.001)	(0.009)	(0.071)	(0.110)	(0.430)	(0.241)	(0.028)	(0.053)	(0.022)	(0.080)	(0.063)
Government Effectiveness	0.046	0.047*	0.037***	0.013**	0.141***	0.000	0.009***	0.096**	0.884***	0.221	1.141	0.332	0.173**	0.300***	0.092*	0.202	0.147
	(0.031)	(0.027)	(0.014)	(0.006)	(0.051)	(0.021)	(0.003)	(0.044)	(0.307)	(0.192)	(1.119)	(0.458)	(0.075)	(0.113)	(0.056)	(0.281)	(0.180)
Regulatory Quality	0.003	-0.005	0.022**	0.012**	-0.010	-0.012	0.002*	0.059**	-0.137	-0.079	0.384	0.218	-0.069	0.032	-0.014	-0.180	0.125
	(0.023)	(0.020)	(0.010)	(0.005)	(0.036)	(0.017)	(0.001)	(0.029)	(0.187)	(0.156)	(0.626)	(0.396)	(0.055)	(0.098)	(0.043)	(0.223)	(0.137)
Rule of Law	-0.025	-0.026	-0.045***	-0.019***	-0.042	0.034	-0.006***	-0.102**	-0.542**	-0.006	-0.577	0.040	-0.044	-0.171	0.013	0.030	-0.290
	(0.032)	(0.029)	(0.016)	(0.007)	(0.050)	(0.023)	(0.002)	(0.044)	(0.214)	(0.182)	(0.842)	(0.516)	(0.071)	(0.119)	(0.062)	(0.228)	(0.197)
Control of Corruption	0.012	0.003	0.018*	0.008*	-0.003	-0.007	0.000	0.007	0.151	0.306	-0.253	-0.289	0.062	0.112	-0.028	0.226	0.310*
	(0.022)	(0.020)	(0.010)	(0.005)	(0.035)	(0.015)	(0.001)	(0.022)	(0.179)	(0.327)	(0.544)	(0.351)	(0.055)	(0.100)	(0.039)	(0.284)	(0.166)
Constant	0.951***	0.928***	0.985***	0.991***	0.909***	0.984***	0.996***	1.008***	0.836***	1.142***	0.137	0.315	0.843***	0.504***	0.827***	1.068***	1.000***
	(0.021)	(0.018)	(0.008)	(0.004)	(0.032)	(0.015)	(0.001)	(0.022)	(0.152)	(0.362)	(0.663)	(0.322)	(0.049)	(0.084)	(0.037)	(0.337)	(0.152)
Observations	105	106	102	107	106	109	100	101	86	104	57	59	102	108	114	99	109
Number of efficient DMUs	15	14	18	13	14	11	20	19	19	16	12	11	18	12	6	21	11
Model degrees of freedom	8	8	8	8	8	8	8	8	8	8	8	8	8	8	8	8	8
Model chi-squared	13.723	8.591	15.819	17.531	19.95	31.85	16.18	8.007	12.549	4.134	2.07	3.484	22.075	38.371	21.828	3.74	11.398
Model significance, p-value	0.089	0.378	0.045	0.025	0.011	0	0.04	0.433	0.128	0.845	0.979	0.9	0.005	0	0.005	0.88	0.18
Number of bootstrap repetitions	1,000	1,000	1,000	1,000	1,000	1,000	1,000	1,000	1,000	1,000	1,000	1,000	1,000	1,000	1,000	1,000	1,000
**LAC**																	
OOP health expenditure as % of CHE	0.000	-0.002	0.000	0.000	-0.002	-0.002	0.000	0.001***	-0.005**	0.002*	0.004	-0.001	0.000	-0.003	0.000	0.000	0.005***
	(0.001)	(0.002)	0.000	0.000	(0.002)	(0.001)	0.000	0.000	(0.002)	(0.001)	(0.006)	(0.004)	(0.001)	(0.003)	(0.004)	(0.002)	(0.002)
Hospital beds per 1.000 people	0.006	-0.004	0.005	0.001	0.021	0.001	0.000	0.005	0.025	-0.030*	0.415*	-0.193	0.002	0.041*	-0.043	-0.011	0.025
	(0.014)	(0.015)	(0.006)	(0.004)	(0.019)	(0.011)	(0.001)	(0.005)	(0.020)	(0.016)	(0.214)	(0.144)	(0.015)	(0.024)	(0.038)	(0.020)	(0.021)
Average governance quality																	
Voice and Accountability	0.057	0.044	-0.019	-0.009	-0.092	-0.049	-0.002	-0.053*	-0.429***	0.137	-0.182	-0.048	0.144**	-0.011	-0.039	-0.066	-0.071
	(0.059)	(0.073)	(0.028)	(0.020)	(0.093)	(0.083)	(0.003)	(0.028)	(0.162)	(0.088)	(0.317)	(0.202)	(0.057)	(0.149)	(0.163)	(0.066)	(0.081)
Political Stability & Absence Violence	0.030	0.013	-0.014	-0.010	0.055	0.011	-0.001	-0.002	0.021	-0.069	-0.055	0.063	-0.184***	-0.114	-0.117	0.042	-0.008
	(0.033)	(0.041)	(0.011)	(0.009)	(0.059)	(0.042)	(0.001)	(0.012)	(0.052)	(0.046)	(0.218)	(0.110)	(0.038)	(0.103)	(0.120)	(0.049)	(0.057)
Government Effectiveness	0.027	0.025	0.014	0.001	-0.008	-0.023	0.000	0.002	0.043	0.084*	-0.255	-0.072	0.055	0.226**	0.094	0.015	-0.022
	(0.035)	(0.045)	(0.011)	(0.007)	(0.057)	(0.046)	(0.001)	(0.010)	(0.074)	(0.048)	(0.267)	(0.138)	(0.042)	(0.092)	(0.144)	(0.068)	(0.054)
Regulatory Quality	0.043	0.093**	0.009	0.012	0.182***	0.102**	0.002	0.030*	0.034	-0.017	0.291	0.005	0.016	-0.002	0.031	0.033	0.041
	(0.042)	(0.044)	(0.011)	(0.011)	(0.064)	(0.044)	(0.002)	(0.016)	(0.052)	(0.042)	(0.201)	(0.129)	(0.061)	(0.087)	(0.134)	(0.050)	(0.052)
Rule of Law	-0.112**	-0.143**	-0.001	-0.015	-0.163**	-0.069	0.000	-0.025*	0.240**	-0.033	0.445	0.595*	-0.049	-0.007	-0.239	-0.012	-0.075
	(0.047)	(0.056)	(0.017)	(0.013)	(0.077)	(0.059)	(0.002)	(0.015)	(0.098)	(0.054)	(0.346)	(0.245)	(0.061)	(0.114)	(0.172)	(0.064)	(0.073)
Control of Corruption	-0.035	0.000	0.011	0.037**	0.011	0.024	0.003	0.077***	0.048	0.069	-0.697	0.091	0.039	-0.086	0.185*	0.041	0.190***
	(0.033)	(0.043)	(0.015)	(0.019)	(0.043)	(0.033)	(0.003)	(0.027)	(0.061)	(0.053)	(0.517)	(0.229)	(0.031)	(0.063)	(0.102)	(0.053)	(0.066)
Constant	0.888***	0.999***	0.993***	1.002***	0.947***	1.019***	1.001***	1.000***	1.342***	0.889***	-0.122	1.571***	0.869***	0.854***	0.852***	0.984***	0.794***
	(0.069)	(0.071)	(0.023)	(0.019)	(0.084)	(0.066)	(0.003)	(0.022)	(0.138)	(0.077)	(0.701)	(0.404)	(0.056)	(0.132)	(0.154)	(0.076)	(0.073)
Observations	15	18	17	19	20	19	17	18	17	21	14	16	17	20	23	20	20
Number of efficient DMUs	11	8	9	7	6	7	9	8	9	5	9	6	9	6	3	6	6
Model degrees of freedom	8	8	8	8	8	8	8	8	8	8	8	8	8	8	8	8	8
Model chi-squared	10.098	12.221	4.971	5.974	10.191	7.836	3.131	12.184	10.486	33.148	19.189	16.351	36.543	18.268	10.031	4.589	12.75
Model significance, p-value	0.258	0.142	0.761	0.65	0.252	0.45	0.926	0.143	0.233	0	0.014	0.038	0	0.019	0.263	0.8	0.121
**MICS**																	
OOP health expenditure as % of CHE	0.001	0.001	0.000	0.000	0.001	-0.001	0.000*	0.000	0.002	0.004	0.010	0.009	0.002*	0.001	0.000	0.004	0.002
	(0.001)	(0.001)	0.000	0.000	(0.001)	0.000	0.000	0.000	(0.002)	(0.003)	(0.019)	(0.011)	(0.001)	(0.002)	(0.001)	(0.003)	(0.002)

Hospital beds per 1.000 people	0.001	0.002	0.007**	0.002**	-0.018**	-0.015***	0.003**	0.021	0.230***	0.022	0.642	0.549	0.034***	0.140***	-0.028***	0.014	0.031
	(0.005)	(0.005)	(0.003)	(0.001)	(0.008)	(0.003)	(0.001)	(0.015)	(0.066)	(0.022)	(0.579)	(0.377)	(0.013)	(0.028)	(0.007)	(0.027)	(0.026)
Average governance quality																	
Voice and Accountability	0.013	0.011	0.003	-0.001	-0.011	-0.017	0.001	0.007	0.136	-0.298**	0.296	0.283	0.027	0.037	0.002	0.066	-0.049
	(0.016)	(0.020)	(0.006)	(0.002)	(0.032)	(0.012)	(0.001)	(0.015)	(0.094)	(0.130)	(0.449)	(0.342)	(0.039)	(0.057)	(0.025)	(0.080)	(0.079)
Political Stability & Absence Violence	-0.012	-0.011	-0.005	-0.001	0.005	0.002	-0.001	0.010	-0.064	0.140**	-0.437	-0.321	-0.024	-0.106**	0.017	-0.023	0.063
	(0.015)	(0.017)	(0.005)	(0.002)	(0.027)	(0.010)	(0.001)	(0.013)	(0.060)	(0.067)	(0.457)	(0.275)	(0.031)	(0.048)	(0.023)	(0.072)	(0.063)
Government Effectiveness	0.032	0.058	0.032**	0.008	0.175**	-0.008	0.010**	0.086	0.753***	0.126	1.141	0.332	0.163*	0.240**	0.061	0.109	0.125
	(0.036)	(0.045)	(0.016)	(0.005)	(0.076)	(0.024)	(0.005)	(0.063)	(0.234)	(0.133)	(1.090)	(0.707)	(0.090)	(0.104)	(0.054)	(0.220)	(0.168)
Regulatory Quality	0.002	-0.003	0.017	0.010**	-0.011	0.002	0.002	0.057	-0.089	-0.012	0.384	0.218	-0.070	0.012	-0.022	-0.208	0.117
	(0.029)	(0.035)	(0.011)	(0.004)	(0.051)	(0.019)	(0.002)	(0.039)	(0.151)	(0.120)	(0.732)	(0.463)	(0.068)	(0.094)	(0.044)	(0.159)	(0.141)
Rule of Law	-0.042	-0.045	-0.043**	-0.018***	-0.077	0.009	-0.007**	-0.103	-0.488***	-0.020	-0.577	0.040	-0.051	-0.157	-0.031	0.031	-0.263
	(0.038)	(0.048)	(0.018)	(0.006)	(0.069)	(0.027)	(0.003)	(0.068)	(0.177)	(0.153)	(0.927)	(0.447)	(0.085)	(0.117)	(0.059)	(0.191)	(0.196)
Control of Corruption	0.019	0.018	0.016	0.007*	-0.004	0.006	0.000	0.000	0.205	0.475**	-0.253	-0.289	0.011	0.046	-0.056	0.152	0.213
	(0.028)	(0.035)	(0.011)	(0.004)	(0.055)	(0.019)	(0.002)	(0.026)	(0.151)	(0.216)	(0.654)	(0.465)	(0.066)	(0.097)	(0.042)	(0.153)	(0.171)
Constant	0.928***	0.951***	0.983***	0.990***	0.938***	1.007***	0.995***	1.001***	0.864***	1.205***	0.137	0.315	0.764***	0.434***	0.794***	0.961***	0.926***
	(0.026)	(0.031)	(0.009)	(0.004)	(0.051)	(0.020)	(0.002)	(0.032)	(0.133)	(0.184)	(0.619)	(0.367)	(0.057)	(0.086)	(0.036)	(0.160)	(0.141)
Observations	70	75	75	77	75	77	70	73	65	75	57	59	72	76	83	72	78
Number of efficient DMUs	17	12	12	10	12	10	17	14	16	12	12	11	15	11	4	15	9
Model degrees of freedom	8	8	8	8	8	8	8	8	8	8	8	8	8	8	8	8	8
Model chi-squared	3.585	3.747	10.592	14.459	8.728	31.079	7.205	3.948	16.209	10.455	1.468	2.331	13.502	31.63	26.208	3.703	6.86
Model significance, p-value	0.893	0.879	0.226	0.071	0.366	0	0.515	0.862	0.039	0.235	0.993	0.969	0.096	0	0.001	0.883	0.552
**OECD**																	
OOP health expenditure as % of CHE	-0.001	0.000	0.000	0.000	0.000	0.000	0.000	0.001**	-0.005	0.001	0.003	0.000	-0.001	-0.003*	0.000	0.001	0.002
	(0.001)	(0.001)	0.000	0.000	(0.001)	(0.001)	0.000	(0.001)	(0.005)	(0.001)	(0.006)	(0.003)	(0.001)	(0.002)	(0.002)	(0.001)	(0.002)
Hospital beds per 1.000 people	-0.005	-0.002	0.005**	0.005*	-0.004	-0.004	0.001**	0.010**	0.050	0.005	0.388**	-0.120	-0.002	0.002	-0.013	0.001	0.005
	(0.003)	(0.003)	(0.002)	(0.003)	(0.005)	(0.003)	0.000	(0.004)	(0.031)	(0.007)	(0.189)	(0.106)	(0.004)	(0.011)	(0.010)	(0.006)	(0.010)
Average governance quality																	
Voice and Accountability	0.015	0.026	-0.004	-0.001	-0.009	-0.005	-0.004**	-0.020	-0.365	0.091*	-0.146	-0.172	0.070**	-0.019	-0.025	-0.052	0.037
	(0.027)	(0.025)	(0.013)	(0.010)	(0.049)	(0.030)	(0.002)	(0.015)	(0.260)	(0.055)	(0.284)	(0.156)	(0.030)	(0.087)	(0.095)	(0.040)	(0.095)
Political Stability & Absence Violence	-0.004	-0.006	-0.008	-0.005	-0.012	-0.011	0.000	0.002	0.098	-0.051	-0.043	0.045	-0.075***	-0.076	-0.076	0.041	-0.064
	(0.016)	(0.014)	(0.008)	(0.006)	(0.029)	(0.016)	(0.001)	(0.009)	(0.108)	(0.033)	(0.222)	(0.114)	(0.018)	(0.061)	(0.057)	(0.025)	(0.063)
Government Effectiveness	0.038	0.039*	0.004	0.001	0.024	0.016	0.000	0.000	0.068	0.079	-0.259	-0.025	0.045	0.272***	0.121	0.019	-0.073
	(0.027)	(0.024)	(0.008)	(0.009)	(0.046)	(0.029)	(0.001)	(0.011)	(0.155)	(0.052)	(0.257)	(0.131)	(0.029)	(0.090)	(0.107)	(0.050)	(0.101)
Regulatory Quality	0.029	0.036**	0.012	0.011	0.054	0.012	0.002**	0.027**	-0.013	0.037	0.279	0.046	0.029	-0.015	0.011	0.021	0.007
	(0.020)	(0.018)	(0.007)	(0.011)	(0.033)	(0.020)	(0.001)	(0.012)	(0.169)	(0.037)	(0.194)	(0.123)	(0.024)	(0.063)	(0.076)	(0.030)	(0.066)
Rule of Law	-0.042	-0.070**	-0.013	-0.019	-0.059	-0.017	-0.001	-0.038**	0.349*	-0.076	0.453	0.524**	-0.071**	-0.039	-0.115	-0.001	-0.047
	(0.029)	(0.027)	(0.012)	(0.013)	(0.052)	(0.031)	(0.001)	(0.017)	(0.206)	(0.057)	(0.335)	(0.227)	(0.034)	(0.102)	(0.112)	(0.048)	(0.109)
Control of Corruption	-0.008	-0.001	0.017**	0.021*	0.022	0.006	0.004**	0.049***	-0.185	-0.002	-0.700	0.150	0.031*	-0.033	0.094	0.027	0.164**
	(0.018)	(0.016)	(0.009)	(0.011)	(0.028)	(0.017)	(0.002)	(0.019)	(0.114)	(0.036)	(0.519)	(0.203)	(0.018)	(0.051)	(0.063)	(0.033)	(0.074)
Constant	0.981***	0.945***	0.989***	0.987***	0.930***	0.947***	1.001***	0.965***	1.394***	0.840***	-0.074	1.462***	0.905***	0.951***	0.829***	0.948***	0.917***
	(0.028)	(0.025)	(0.011)	(0.009)	(0.040)	(0.025)	(0.002)	(0.017)	(0.342)	(0.050)	(0.662)	(0.323)	(0.028)	(0.081)	(0.085)	(0.043)	(0.084)
Observations	48	50	48	50	53	52	50	48	38	51	15	17	47	50	55	47	49
Number of efficient DMUs	12	10	12	10	7	8	10	12	13	9	9	6	13	10	5	13	11
Model degrees of freedom	8	8	8	8	8	8	8	8	8	8	8	8	8	8	8	8	8
Model chi-squared	17.638	12.926	10.435	7.39	8.239	4.976	12.554	9.203	7.6	24.82	20.366	18.288	55.525	35.324	12.359	20.509	7.957
Model significance, p-value	0.024	0.114	0.236	0.495	0.41	0.76	0.128	0.325	0.473	0.002	0.009	0.019	0	0	0.136	0.009	0.438

Source: Author's calculations.

Notes: Simar-Wilson models estimated with 1,000 bootstrap replications. Robust standard errors in parenthesis. *p<0.1, **p<0.5, **p<0.01.
