# Supplementary material for: Efficiency of health systems in middle-income countries and determinants of efficiency in Latin America and the Caribbean
Source: PLoS One. 2024 Sep 5;19(9):e0309772. doi: 10.1371/journal.pone.0309772 (PMC11376550; doi:10.1371/journal.pone.0309772)
Supplement: S1 Appendix — (PDF) [file pone.0309772.s015.pdf]

S1 Appendix

Sample of countries

We used data from countries in Latin America and the Caribbean (LAC), Organisation for Economic Co-operation and Development (OECD), and middle-income countries (MICS).

For LAC, we used data from 26 countries including Argentina (ARG), Bahamas (BHS), Barbados (BRB), Belize (BLZ), Bolivia (BOL), Brazil (BRA), Chile (CHL), Colombia (COL), Costa Rica (CRI), Dominican Republic (DOM), Ecuador (ECU), El Salvador (SLV), Guatemala (GTM), Guyana (GUY), Haiti (HTI), Honduras (HND), Jamaica (JAM), Mexico (MEX), Nicaragua (NIC), Panama (PAN), Paraguay (PRY), Peru (PER), Suriname (SUR), Trinidad and Tobago (TTO), Uruguay (URY), and Venezuela (VEN).

For OECD, we used data from 34 countries including Australia (AUS), Austria (AUT), Belgium (BEL), Canada (CAN), Switzerland (CHE), Czech Republic (CZE), Germany (DEU), Denmark (DNK), Spain (ESP), Estonia (EST), Finland (FIN), France (FRA), United Kingdom (GBR), Greece (GRC), Hungary (HUN), Ireland (IRL), Iceland (ISL), Israel (ISR), Italy (ITA), Japan (JPN), Korea (KOR), Lithuania (LTU), Luxembourg (LUX), Latvia (LVA), Netherlands (NLD), Norway (NOR), New Zealand (NZL), Poland (POL), Portugal (PRT), Slovak Republic (SVK), Slovenia (SVN), Sweden (SWE), Turkey (TUR), and United States (USA). We also included data from 4 OECD LAC countries: Chile (CHL), Colombia (COL), Costa Rica (CRI), and Mexico (MEX).

For MICS, we used data from 49 lower-middle-income and 40 upper-middle-income. For lower MICS we included Angola (AGO), Benin (BEN), Bangladesh (BGD), Bhutan (BTN), CÃ´te d'Ivoire (CIV), Cameroon (CMR), Congo, Rep. (COG), Comoros (COM), Cabo Verde (CPV), Djibouti (DJI), Algeria (DZA), Egypt, Arab Rep. (EGY), Micronesia, Fed. Sts. (FSM), Ghana (GHA), Indonesia (IDN), India (IND), Iran, Islamic Rep. (IRN), Kenya (KEN), Kyrgyz Republic (KGZ), Cambodia (KHM), Kiribati (KIR), Lao PDR (LAO), Lebanon (LBN), Sri Lanka (LKA), Lesotho (LSO), Morocco (MAR), Myanmar (MMR), Mongolia (MNG), Mauritania (MRT), Nigeria (NGA), Nepal (NPL), Pakistan (PAK), Philippines (PHL), Papua New Guinea (PNG), West Bank and Gaza (PSE), Senegal (SEN), Solomon Islands (SLB), Sao Tome and Principe (STP), Eswatini (SWZ), Tajikistan (TJK), Timor-Leste (TLS), Tunisia (TUN), United Republic of Tanzania (TZA), Ukraine (UKR), Uzbekistan (UZB), Viet Nam (VNM), Vanuatu (VUT), Samoa (WSM), and Zimbabwe (ZWE). For upper MICS we included Albania (ALB), Armenia (ARM), American Samoa (ASM), Azerbaijan (AZE), Bulgaria (BGR), Bosnia and Herzegovina (BIH), Belarus (BLR), Botswana (BWA), China (CHN), Cuba (CUB), Dominica (DMA), Fiji (FJI), Gabon (GAB), Georgia (GEO), Equatorial Guinea (GNQ), Grenada (GRD), Iraq (IRQ), Jordan (JOR), Kazakhstan (KAZ), Libya (LBY), St. Lucia (LCA), Republic of Moldova (MDA), Maldives (MDV), Marshall Islands (MHL), North Macedonia (MKD), Montenegro (MNE), Mauritius (MUS), Malaysia (MYS), Namibia (NAM), Palau (PLW), Russian Federation (RUS), Serbia (SRB), Thailand (THA), Turkmenistan (TKM), Tonga (TON), Turkey (TUR), Tuvalu (TUV), St. Vincent and the Grenadines (VCT), Kosovo (XKX), and South Africa (ZAF). We also included 19 LAC MICS countries, that includes all countries but Bahamas (BHS), Chile (CHL), Panama (PAN), Trinidad and Tobago (TTO), Uruguay (URY), and Venezuela (VEN).
